# Bcl‐2 inhibitors enhance FGFR inhibitor‐induced mitochondrial‐dependent cell death in FGFR2‐mutant endometrial cancer

**DOI:** 10.1002/1878-0261.12422

**Published:** 2019-01-18

**Authors:** Leisl M. Packer, Samantha J. Stehbens, Vanessa F. Bonazzi, Jennifer H. Gunter, Robert J. Ju, Micheal Ward, Michael G. Gartside, Sara A. Byron, Pamela M. Pollock

**Affiliations:** ^1^ School of Biomedical Science Institute of Health & Biomedical Innovation Queensland University of Technology located within the Translational Research Institute Brisbane Australia; ^2^ Mater‐UQ located within the Translational Research Institute Brisbane Australia; ^3^ Cancer and Cell Biology Division Translational Genomics Research Institute Phoenix AZ USA; ^4^ Present address: University of Queensland Diamantina Institute located within the Translational Research Institute Brisbane Qld Australia

**Keywords:** ABT263, BGJ398, cell death, endometrial cancer, FGFR2 inhibitor

## Abstract

Endometrial cancer is the most commonly diagnosed gynaecological malignancy. Unfortunately, 15–20% of women demonstrate persistent or recurrent tumours that are refractory to current chemotherapies. We previously identified activating mutations in fibroblast growth factor receptor 2 (FGFR2) in 12% (stage I/II) to 17% (stage III/IV) endometrioid ECs and found that these mutations are associated with shorter progression‐free and cancer‐specific survival. Although FGFR inhibitors are undergoing clinical trials for treatment of several cancer types, little is known about the mechanism by which they induce cell death. We show that treatment with BGJ398, AZD4547 and PD173074 causes mitochondrial depolarization, cytochrome c release and impaired mitochondrial respiration in two FGFR2‐mutant EC cell lines (AN3CA and JHUEM2). Despite this mitochondrial dysfunction, we were unable to detect caspase activation following FGFR inhibition; in addition, the pan‐caspase inhibitor Z‐VAD‐FMK was unable to prevent cell death, suggesting that the cell death is caspase‐independent. Furthermore, while FGFR inhibition led to an increase in LC3 puncta, treatment with bafilomycin did not further increase lipidated LC3, suggesting that FGFR inhibition led to a block in autophagosome degradation. We confirmed that cell death is mitochondrial‐dependent as it can be blocked by overexpression of Bcl‐2 and/or Bcl‐XL. Importantly, we show that combining FGFR inhibitors with the BH3 mimetics ABT737/ABT263 markedly increased cell death *in vitro* and is more effective than BGJ398 alone *in vivo*, where it leads to marked tumour regression. This work may have implications for the design of clinical trials to treat a wide range of patients with FGFR‐dependent malignancies.

**Abbreviations**
ANOVA: analysis of variance
CICD: caspase‐independent cell death
DMSO: dimethyl sulfoxide
EC: endometrial cancer
ECAR: extracellular acidification rate
ECS: endometrial cancer‐specific survival
FBS: fetal bovine serum
FGFR: fibroblast growth factor receptor
MOMP: mitochondrial outer membrane permeabilization
NSG: NOD scid gamma
NT: nontargeting
OCR: oxygen consumption rate
PFS: progression‐free survival
SD: standard deviation
SEM: standard error of the mean
TMRE: tetramethylrhodamine ethyl ester
TRI: Translational Research Institute

## 1 Introduction

Endometrial cancer (EC) is the most commonly diagnosed malignancy of the female reproductive tract in developed countries. While 75% of ECs are detected early and have a good prognosis, patients who relapse postresection or present with metastatic disease have a very poor prognosis with a median survival of 7–12 months (Stelloo *et al*., 2016). Our laboratory was the first to identify activating mutations in fibroblast growth factor receptor 2 (FGFR2) in endometrioid ECs (Pollock *et al*., 2007). Analysis of FGFR2 mutations in a single institution cohort (*n* = 466) revealed that FGFR2 mutation was associated with shorter progression‐free survival in patients with early‐stage (I/II) disease (Byron *et al*., 2012). Subsequent analysis in a larger multi‐institutional cohort revealed FGFR2 mutations in 12% of stage I/II cases and 17% of stage III/IV cases. In multivariate analysis, FGFR2 mutation was confirmed to be associated with shorter progression‐free survival (PFS) (HR 1.903; 95% CI 1.177 – 3.076; *P* = 0.009) and shorter EC‐specific survival (ECS) (HR 2.013; CI 95% 1.096 – 3.696; *P* = 0.024) in 803 early‐stage cases. Significantly shorter PFS and ECS were also seen in the entire cohort of 970 patients (Jeske *et al*., 2017).

Activation of FGFR1, 2 and 3 has been reported in a diverse range of cancer types (Turner and Grose, 2010a), and *in vitro* studies have revealed both cytostatic and cytotoxic responses to FGFR inhibition in FGFR‐mutant cancer cell lines (Gavine *et al*., 2012; Kunii *et al*., 2008; Lamont *et al*., 2011). FGFR inhibitors have been shown to induce caspase‐3/7 activity, annexin positivity and/or a subG1 peak in various FGFR‐dependent cell lines including SCLC and NSCLC (Pardo *et al*., 2009; Weiss *et al*., 2010), breast cancer (Gavine *et al*., 2012; Sharpe *et al*., 2011; Turner *et al*., 2010c), myeloma (Gavine *et al*., 2012), gastric cancer (Kunii *et al*., 2008; Pearson *et al*., 2016; Xie *et al*., 2013) and EC (Byron *et al*., 2008; Konecny *et al*., 2013; Kwak *et al*., 2015). Although some groups utilize two assays, the induction of apoptosis is often concluded from the presence of caspase activity alone (Pearson *et al*., 2016), a subG1 peak alone (Xie *et al*., 2013) or annexin positivity alone (Gavine *et al*., 2012; Koneczny *et al*., 2015; Kwak *et al*., 2015; Weiss *et al*., 2010). A recent study in lung cancer reported the presence of both caspase‐dependent and caspase‐independent cell death following FGFR inhibition in a single lung cancer cell line (Goke *et al*., 2015a), but no detailed study of the mechanism of cell death has been published in any tumour type.

Although FGFR inhibitors have shown efficacy in preclinical models, the responses in the clinic have been disappointing. Similar to other kinase inhibitors, single‐agent therapy was often not sufficient to cause tumour regression with only transient tumour responses observed (Sequist *et al*., 2014; Soria *et al*., 2014). Prolonged responses have been seen, but these are limited to those patients whose tumours show high‐level amplifications or FGFR2/3 fusions rather than those with activating mutations in FGFR1‐3 (Babina and Turner, 2017). Presumably, the therapeutic response could be enhanced by combination therapies that target additional prosurvival signals within the cell. Therefore, understanding the mechanism of cell death is critical.

As the cell death we have observed in EC occurs in the presence of constitutive PI3K activity (Byron *et al*., 2008), and is therefore likely to be different than in those cancers where FGFR inhibitors block both the MAPK and PI3K downstream signalling pathways (Chell *et al*., 2013; Gavine *et al*., 2012; Pearson *et al*., 2016; Turner *et al*., 2010b), we sought to investigate in detail the molecular mechanism underlying this cell death. We have employed multiple FGFR inhibitors (PD173074, AZD4547, BGJ398) and several EC cell lines carrying different FGFR2 mutations. Our results show that FGFR inhibition induces mitochondrial‐dependent cell death that, when combined with BH3 mimetics (such as ABT263), leads to enhanced cell death, likely through caspase activation. These results open up the possibility of a clinical trial testing this combination in EC as well as other cancers where caspase‐dependent cell death has been reported.

## 2 Materials and methods

### 2.1 Cell lines

AN3CA, JHUEM2 and MFE296 were obtained from ATCC, Riken Cell Bank and ECACC, respectively. AN3CA, JHUEM2 and MFE296 were authenticated by STR profiling at the sequencing facility of The QIMR Berghofer Medical Research Institute in 2016 and 2018 and passaged less than 20 times since authentication. AN3CA and MFE296 were grown in MEM‐alpha and JHUEM2 cells in 1 : 1 DMEM:Ham's F12, supplemented with 10% fetal bovine serum (FBS), 1% penicillin/streptomycin and 0.1 mm nonessential amino acids. According to the Cancer Cell Line Encyclopedia, the cell lines harbour the following mutations: AN3CA FGFR2^N550K,K310R^, JHUEM2 FGFR2^C383R^ and MFE296 FGFR2^N550K^. BGJ398, AZD4547, PD173074, ABT737, ABT263, paclitaxel and necrostatin‐1 were purchased from Selleckchem; Z‐VAD‐FMK, bafilomycin and staurosporine from LClabs; and actinomycin from Sigma (Castle Hill, NSW, Australia).

### 2.2 Annexin positivity

Floating and attached cells were collected and analysed for Annexin and PI staining according to the manufacturer's instructions (FITC Annexin V Apoptosis Detection Kit II, BD Biosciences, North Ryde, NSW, Australia) using the BD LSR II and flowjo v10.7 (Ashland, OR, USA). An equal number of cells were stained to ensure that Annexin V levels could be accurately assessed and compared across samples. All annexin‐positive cells were counted (whether PI positive or negative).

### 2.3 Cell survival assay

Cells (600–1000) were seeded in 6‐well plates and the following day treated with DMSO or inhibitors for 72 h. Cells were washed thrice in PBS and grown in full‐growth medium for 10–16 days, fixed with methanol and stained with crystal violet (0.1% in 25% methanol). Colonies were counted and plotted as a percentage of the DMSO control.

### 2.4 Western blot, immunoprecipitation and antibodies

Proteins were harvested using RIPA buffer (50 mm Tris, pH 7.4, 150 mm NaCl, 1 mm EDTA, 1% IGEPAL, 0.1% SDS, 0.5% sodium deoxycholate, 1 mm sodium orthovanadate, 1 mm NaF, 1 mm PMSF, 10 μg·mL^−1^ aprotinin and leupeptin). Western blotting was performed using standard protocols. To stimulate FGFR2, cells were treated with 10 ng·mL^−1^ FGF10 (R&D systems, Noble Park, VIC, Australia) and 5 μg·mL^−1^ heparin (Sigma) for 10 min prior to collection of lysate. FGFR2 immunoprecipitation was performed as described in Packer *et al*. (2017). Bim IP was performed as described in Packer *et al*. (2017). Normalized IP fold changes were calculated as follows:

$$\frac{\text{Interacting}\;{\text{protein}}_{\mathrm{treatment}}}{{\text{Bim}}_{\mathrm{treatment}}÷{\text{Bim}}_{\mathrm{DMSO}}}÷\frac{\text{Interacting}\;{\text{protein}}_{\mathrm{DMSO}}}{{\text{Bim}}_{\mathrm{DMSO}}÷{\text{Bim}}_{\mathrm{DMSO}}}$$

The following antibodies were used: AIF (#5318), pAKT (ser473) (#4060) AKT (#2920), ATG3 (#3415), ATG7 (#8558), ATG12 (#4180), Bcl‐XL (#2764), BIM (#2933), BID (#2002), caspase‐3 (#9662), caspase‐7 (#9502), Cox IV (#4844), cytochrome c (#4272), ERK1/2 (#4695), pFRS2a (Tyr436) (#3861), LC3 (#2775) and Smac/diablo (#2954) from Cell Signaling Technology; pERK (Thr202/Tyr204) (M8159), p53 (P8999) and tubulin (T9026) from Sigma; PUMA (PC686) and Bcl‐2 (OP60) from Calbiochem; Bcl‐XL (#610746) and Mcl‐1 from BD Biosciences; EndoG (AB9647) from Abcam; FGFR2 (sc‐122), FRS2 (sc‐8318) and Tom20 (sc‐11415) from Santa Cruz Biotechnology; and HTRA2/OMI (AF1458) from R&D Systems.

### 2.5 Flow cytometry measurement of cytochrome c release

We followed the method described in Christensen *et al*. (2013). Samples were analysed on the BD LSR II and analysed using flowjo v10.7.

### 2.6 TMRE staining

Cells (AN3CA, JHUEM2) were plated onto glass‐bottom dishes (Cellvis 4‐Chamber 35‐mm Glass Bottom Dish with 20‐mm microwell, #1.5 cover glass, D35C4‐20‐1.5‐N) or #1.5 coverslips (immunofluorescence), 24 h prior to treatment with FGFR inhibitors for 48 h. Samples for immunofluorescence were fixed in 4% PFA in BRB80 buffer (80 mm K‐PIPES, pH 6.8, 1 mm MgCl_2_, 1 mm EGTA) for 20 min at room temperature, washed three times with PBS and permeabilized with 0.25% Triton X‐100 in PBS for 5 min. Samples plated on glass‐bottom dishes were processed for live‐cell analysis of mitochondrial function. Control cells were pretreated with FCCP (10 μm) for 10 min prior to labelling to ensure that TMRE (Tetramethylrhodamine, Ethyl Ester, Perchlorate, ThermoFisher T669) is below quenching level. Mitochondria were labelled with MitoTracker Green FM (100 nm) and TMRE (50 nm) as per the manufacturer's instructions. In brief, cells were incubated in full‐growth media containing MitoTracker Green FM (ThermoFisher M7514) for 15 min at 37 °C. Cells were rinsed three times with media to remove unbound dye before incubating with TMRE for 15 min at 37 °C and rinsed three times in full‐growth media. Cells were imaged immediately on a spinning disc confocal microscope (>40 min) (Stehbens *et al*., 2012), in media supplemented with 20 mm HEPES (pH 7.5) (GIBCO).

### 2.7 LC3 staining

For LC3 staining, samples were fixed in ice‐cold methanol for 5 min at room temperature, followed by rehydration in PBS and processing for immunofluorescence. Serum starvation in 0.5% FBS was included as a control for induction of LC3 puncta. Fixed samples were rinsed 3–5 times in blocking buffer (2% BSA, 0.1% Triton X‐100, 0.1% NaN_3_ in PBS). Samples were incubated with LC3 diluted in blocking buffer for 1 h at room temperature. Samples were rinsed 3–5 times in blocking buffer for 5–10 min each before incubating in secondary antibodies (1 : 500) for 45 min at room temperature. Secondary antibodies and phalloidin were diluted in blocking buffer. Samples were rinsed 3–5 times in PBS, 5–10 min each, and then mounted in Mowiol mounting medium (0.1 M Tris/HCl, pH 8.5, 25% glycerol, 10% Mowiol 4‐88; 475904, Calbiochem, 2% DABCO; D2522, Sigma). All secondary fluorescently labelled antibodies were highly cross‐absorbed secondary antibodies from Jackson ImmunoResearch, Alexa Fluor^®^ 488 Anti‐Rabbit (711‐545‐152) and Rhodamine (TRITC) Anti‐Mouse (715‐025‐151), used at 1 : 500.

### 2.8 TMRE and LC3 image analysis

Mitochondria, TMRE and LC3 puncta were imaged on a spinning disc confocal microscope, details of which are published elsewhere (Stehbens *et al*., 2012). Image processing and analysis was performed in NIS Elements (Nikon). Generally, for display purposes image contrast was linearly adjusted on the 14‐ or 16‐bit raw data, and images were low‐pass‐filtered in NIS Elements (Detail level: 2) and processed with an unsharp mask filter (Power: 0.5; Area: 7). To quantify TMRE staining, the total mitochondrial region of interest (ROI) was defined by thresholding to create a binary mask of the 488 channel (MitoTracker Green FM) in NIS Elements. The binary mask was converted to ROI, and ROI mean pixel intensities were measured for both the 594 and 488 channels and expressed as a ratio of TMRE/MitoTracker. To quantify LC3 puncta, images were thresholded for bright objects using the Object Count tool in NIS Elements. The number of LC3 objects/puncta was normalized to the number of nuclei per field of view.

### 2.9 Subcellular fractionation

Mitochondrial and cytosolic fractions were extracted according to Holden and Horton (2009). Briefly, cells were pelleted, washed in ice‐cold PBS, lysed in 400 μL cold cytosolic buffer (150 mm NaCl, 50 mm HEPES, pH 7.4, 25 μg·mL^−1^ digitonin, protease and phosphatase inhibitors) for 10 min and then centrifuged at 376 *g*. Supernatant was collected as the cytosolic fraction. The cell pellet was washed in PBS and resuspended by vortexing in 200 μL cold NP‐40 buffer (150 mm NaCl, 50 mm HEPES, pH 7.4, 1% NP‐40, protease and phosphatase inhibitors), placed on ice for 30 min and centrifuged at 7000 RCF. The supernatant (containing mitochondria) was collected.

### 2.10 Lentiviral shRNA knockdown

The following pLKO.1‐puro lentiviral shRNA plasmids were used in this study: pKLO.1 nontargeting (NT) shRNA (puro) plasmid #1864 (Addgene, Waterton, MA, USA), Bim #1 TRCN0000356031, Bim #2 TRCN0000001051 (Sigma), EndoG #1 TRCN0000039643, EndoG #2 TRCN0000039645 (Dharmacon, Millennium Science, Mulgrave, VIC, Australia), AIF #1 TRCN0000229860, AIF #2 TRCN0000064489 (sigma), ATG3 TRCN0000149597, ATG7.1 TRCN0000007584, ATG7.2 TRCN0000007587 and ATG12 TRCN0000007394 (kindly provided by Jay Debnath, UCSF).

To generate lentiviral particles, 8 × 10^6^ HEK293FT cells were seeded in a T75 flask. The next day, cells were transfected with the following mixture 500 μL Opti‐MEM, 26 μL lipofectamine 2000, packaging vectors 0.5 μg pTAT, 7.1 μg pNHP, 2.8 μg pHEF‐VSVG and 3.5 μg pLKO.1 plasmid. Virus was collected at 48, 72 and 96 h post‐transfection. To establish stable knockdown of genes, 500 000 cells were seeded in a T25 flask. The following day, cells were infected with 1 mL of virus, 4 μg·mL^−1^ polybrene in 4 mL media. Puromycin Dihydrochloride (1–2 μg·mL^−1^, Invtrogen, ThermoFisher, Melbourne, VIC, Australia, A11138‐03) selection took place 48 h postinfection.

### 2.11 Overexpression plasmids

Bcl‐2 and Bcl‐XL constructs were obtained from Origene (SC125546 and SC127825) and subsequently subcloned into pEF1αIRES.neo.3. AN3CA and JHUEM2 cells were transfected with FuGENE 6 and Lipofectamine 2000 according to standard protocols. Stable expression was selected for in G418.

### 2.12 Measurement of oxygen consumption

Oxygen consumption rate (OCR) was measured with the optical fluorescent oxygen/hydrogen sensor XF24 Seahorse analyser (Agilent, Mulgrave, VIC, Australia). Briefly AN3CA cells (20 000/well) were incubated overnight in 250 μL media. The following day, cells were treated with FGFR inhibitors for 48 h (AN3CA). Cells were then washed into unbuffered DMEM with an adjusted pH of 7.4 according to the manufacturer's instructions. The Seahorse XF Cell Mito Stress Kit was used to measure OCR using the following concentrations: 1.2 μm oligomycin, 1 μm FCCP, 1 μm rotenone and 1 μm antimycin A. All OCR measurements were normalized to cell number [using the CyQUANT Cell Proliferation Assay (ThermoFisher, Melbourne, VIC, Australia)] and used to calculate various mitochondrial parameters following established methods (Brand and Nicholls, 2011).

### 2.13 *In vivo* mouse xenografts

All mice were acclimated for seven days prior to handling. Mice were maintained and handled under aseptic conditions, allowed access to food and water *ad libitum* and maintained under specific pathogen‐free conditions. The mice were closely followed and would be euthanized if they showed signs of ill health or stress, such as inactivity, ruffled fur coat or anorexia.

Five‐week‐old female NSG mice (16–20 g) were purchased from the Australian BioResources (Moss Vale, Australia) and hosted in the pathogen‐free Biological Resource Facility of the Translational Research Institute (Brisbane, Australia). *In vivo* animal studies were performed according to institution‐approved protocols (Translational Research Institute TRI/416/17/AUC), and guidelines for maintenance of animals and endpoint of tumour studies were followed. Xenografts of AN3CA were established by subcutaneously injecting 4 × 10^5^ cells in growth factor‐reduced Matrigel (#354230, BD Biosciences) 1 : 1 with PBS. Perpendicular tumour diameters were measured using Vernier‐scale callipers, and tumour volumes were calculated using the formula [(*x *× *y* ^2^)/2]. AN3CA xenografts were allowed to grow for 10 days, to allow formation of tumours with mean xenograft volume ~150 mm^3^. Mice were then stratified into treatment groups with one tumour per mouse on the basis of their weight and tumour volume. Mice (8/group) were treated for 2 weeks via oral gavage, 6 days on/1 day off, of (a) vehicle control [100 mmol·L^−1^ acetic acid/sodium acetate buffer (pH 4.6)/PEG 300 (1 : 1)]; (b) BGJ398, 20 mg·kg^−1^; (c) ABT263, 100 mg·kg^−1^; or (d) BGJ398+ ABT263. Body weight was recorded for each animal every other day to monitor potential toxicities. Additional animals (4/group) were treated for 7 days, with their final treatment 4 h prior to tumour collection.

### 2.14 Immunohistochemistry and digital image analysis

Cleaved caspase‐3 staining was performed as previously described (Packer *et al*., 2017). After sectioning and staining, tumour sections were digitally scanned at x40 magnification, using an Olympus VS120 slide scanner at the TRI microscopy facility. visiopharm integrator system v2107.2 (Visiopharm A/S, Hoersholm, Denmark) was used to score for cleaved caspase‐3 status. The software was trained to dissociate positive staining from background and to exclude necrotic areas and blood cells. For each sample, the whole section was delimitated and the visiopharm software returned specific area value for each tissue type. The ratio of the area value of the cleaved caspase‐3 positively stained cells to the entire tissue area was then calculated.

### 2.15 Statistical analysis

Annexin positivity, cell survival growth, cytochrome c release and spare respiratory capacity were analysed using one‐way ANOVA with Dunnett's multiple comparison to compare treatments to control. Caspase‐3 staining by IHC was analysed using one‐way ANOVA with Tukey's multiple correction test. LC3 and TMRE staining was analysed using a Kruskal–Wallis one‐way ANOVA with a Dunn's multiple comparison test. To analyse the four treatment conditions of the Bim IP experiments, a two‐way ANOVA with Holm–Sidak multiple comparisons correction tests was used. *P*‐values, calculated with prism (GraphPad, San Diego, CA, USA), are coded by asterisks: <0.05 (*), <0.01 (**), <0.001 (***) and *P* < 0.0001 (****). Differences in xenograft volume between groups were assessed for significance using a repeated two‐way ANOVA with Tukey's multiple comparisons test.

## 3 Results

**Figure 1:**
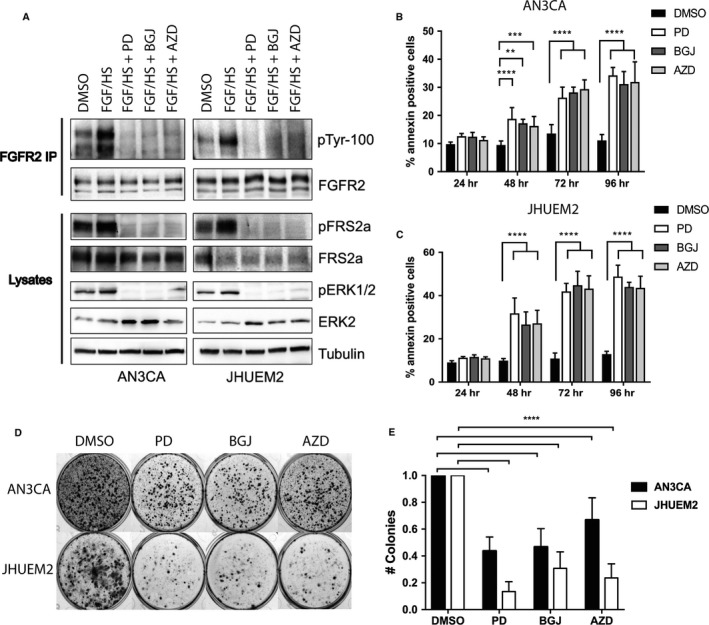
Fibroblast growth factor receptor inhibitors induce cell death, reduce long‐term survival and inhibit the *in vivo* growth of FGFR2‐mutant EC cells. (A) Western blots showing immunoprecipitates (FGFR2 IP) or whole‐cell lysates from AN3CA and JHUEM2 cells cultured overnight in 0.5% FBS with 1‐h treatment with DMSO, 1 μm PD173074 (PD), 300 nm BGJ398 (BGJ) or 300 nm AZD4547 (AZD), with a 10‐min stimulation with 50 ng·mL^−1^ FGF10 and 5 μg·mL^−1^ heparan sulfate (FGF/HS) immediately prior to cell lysis. (B) AN3CA and (C) JHUEM2 cells were treated with the above concentrations of PD, BGJ and AZD for 72 h. Cell death was detected by staining cells with Annexin V. The mean percentage of Annexin V‐positive cells from three independent experiments (each performed in triplicate) is shown along with SD. Data were analysed using a one‐way ANOVA with Dunnett's multiple comparison to compare treatments to control. (D) Clonogenic survival assays in AN3CA and JHUEM2 with the above doses of PD, BGJ and AZD for 72 h. Cells were then cultured for approximately 2 weeks and stained with crystal violet. (E) The mean number of colonies (expressed as a fraction of DMSO) of three independent experiments (each performed in triplicate), error bars represent SD. One‐way ANOVA with Dunnett's multiple comparison to compare treatments to control. *P* <0.01 (**), <0.001 (***), <0.0001 (****).

The FGFR2‐mutant EC cell lines AN3CA (FGFR2^N550K,K610R^) and JHUEM2 (FGFR2^C383R^) were used in this study, due to their sensitivity to FGFR inhibitors. The IC_50_ values of PD173074, BGJ398 and AZD4547 are shown in Table 1. We first confirmed FGFR2 inhibition by PD173074, BGJ398 and AZD4547 in these cells. Figure 1A shows immunoprecipitation of FGFR2 from cells treated with DMSO, cells stimulated with FGF10 (50 ng·mL^−1^) and heparin (5 μg·mL^−1^) for 10 min and cells treated with FGFR inhibitors for 1 h, followed by FGF10/heparin stimulation. Probing the FGFR2‐enriched fraction for phospho‐tyrosine shows activation of FGFR2 by FGF10 stimulation and loss of phosphorylated FGFR2 following inhibitor treatment. This corresponds with the activation of downstream effectors FRS2α and ERK1/2 by FGF10/heparin stimulation and inhibition by PD173074, BGJ398 and AZD4547.

**Table 1:** IC_50_ values for FGFR inhibitors PD173074, BGJ398 and AZD4547 in AN3CA and JHUEM2 cells

Cell line	PD173074 IC_50_ (nm)	BGJ398 IC_50_ (nm)	AZD4547 IC_50_ (nm)
AN3CA	110	30	45
JHUEM2	100	20	30

We next investigated the timing of cell death induced by FGFR inhibition. Cells were treated with 1 μm PD173074, 300 nm BGJ398 and 300 nm AZD4547, and annexin positivity, as measured by flow cytometry, was used to determine the percentage of dead cells (Fig. 1B,C). Unlike many chemotherapy drugs, which induce cell death within 24 h, cell death resulting from FGFR inhibition was delayed. A significant increase in annexin positivity was observed at 48 h (AN3CA PD *P *<* *0.001, BGJ *P *<* *0.01, AZD *P *<* *0.001; JHUEM2; *P *<* *0.001 PD, BGJ and AZD) and 72 and 96 h in both cell lines (*P *<* *0.0001 PD, BGJ and AZD). Both cell lines exhibited 10% annexin staining in the DMSO control, and this increased to 30–35% at 72–96 h of treatment in AN3CA cells and 30–50% in JHUEM2 cells at 48–72 h of treatment with FGFR inhibitors. FGFR inhibition also significantly reduced long‐term cell survival in both cell lines, albeit more potently in JHUEM2 (Fig. 1D–E; *P *<* *0.0001). This is consistent with *in vivo* results showing reduction in tumour growth in AN3CA and JHUEM2 cells treated with BGJ398 (Packer *et al*., 2017).

**Figure 2:**
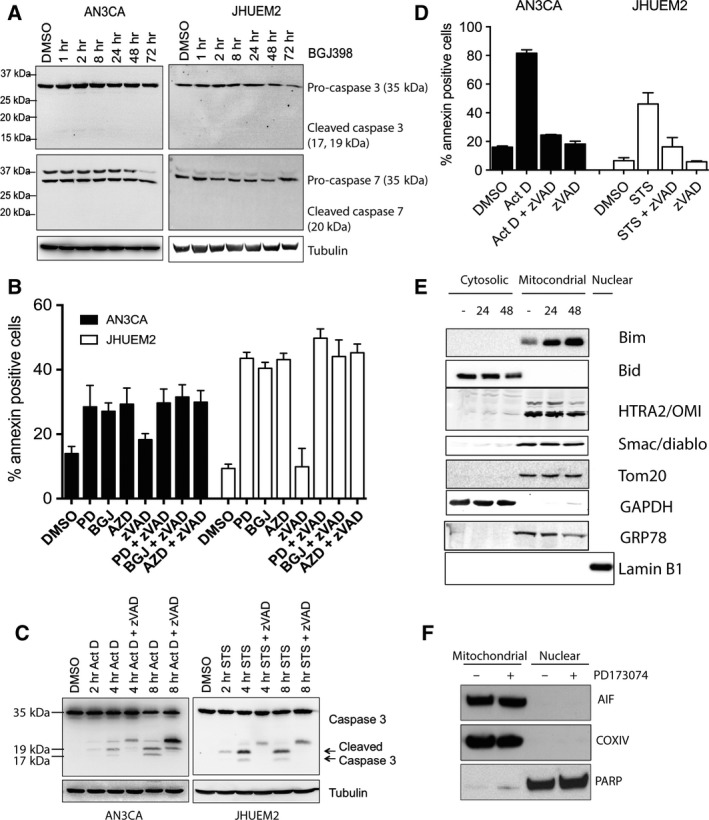
Caspases are not activated by FGFR inhibition in FGFR2‐mutant EC cells. (A) Western blots showing total caspase‐3 and caspase‐7 in response to treatment with 300 nm BGJ398 for up to 72 h. Tubulin serves as a loading control. *Denotes nonspecific band. (B) AN3CA and JHUEM2 cells were pretreated with 100 μm Z‐VAD‐FMK for 1 h prior to the addition of DMSO, 1 μm PD173074 (PD), 300 nm BGJ398 (BGJ) or 300 nm AZD4547 (AZD) for 72 h. Cell death was detected by staining cells with Annexin V. The mean percentage of Annexin V‐positive cells from three independent experiments (each performed in triplicate) is shown along with SD. (C) Western blot showing cleavage of caspase‐3 in AN3CA and JHUEM2 cells treated with 1 μm actinomycin D (Act D) or staurosporine (STS), respectively, for 24 h. (D) Mean percentage of AN3CA and JHUEM2 cells showing Annexin V‐positive staining following treatment with 100 μm Z‐VAD‐FMK alone or 1 h prior to treatment with 1 μm Act D and STS for 24 h. The mean from three independent experiments (each performed in triplicate) is shown along with SD. (E) Western blot showing staining of Bim, Bid, HTRA2/OMI and Smac/diablo in cytosolic and mitochondrial fractions of JHUEM2 cells treated with 300 nm BGJ398 for 24 and 48 h. Tom20 serves as a marker of the mitochondrial fraction, GRP78 as a marker of the ER, Lamin B1 as a marker of the nuclear fraction and GAPDH as a marker of the cytosolic fraction. (F) Western blot showing staining of AIF in mitochondrial and nuclear fractions of AN3CA cells treated with 1 μm PD173074 for 48 h. Cox IV and PARP serve as mitochondrial and nuclear markers, respectively.

The mechanism by which FGFR inhibitors PD173074, BGJ398 and AZD4547 induce cell death in these cells was further investigated. Several groups have reported caspase‐3/7 cleavage following FGFR inhibition in breast and other cancer lines, suggesting a ‘classical’ apoptotic response to FGFR inhibitors (Goke *et al*., 2015a; Hall *et al*., 2016; Pearson *et al*., 2016; Sharpe *et al*., 2011). Unlike these previous reports, we observed no cleavage of caspase‐3 and caspase‐7 following FGFR inhibition in AN3CA or JHUEM2 cells over a 72‐h time course by western blotting (Fig. 2A). To confirm that FGFR inhibitor‐mediated cell death was caspase‐independent, we examined whether cell death could be blocked by the pan‐caspase inhibitor Z‐VAD‐FMK (Fig. 2B). Pretreatment of AN3CA and JHUEM2 with 100 μm Z‐VAD‐FMK did not abrogate cell death following FGFR inhibition in either cell line, suggesting that FGFR inhibitors induce caspase‐independent cell death (CICD) in FGFR2‐mutant EC cells. To ensure that AN3CA and JHUEM2 cells are capable of undergoing caspase‐dependent apoptosis, we treated cells with 1 μm actinomycin D (AN3CA) or 1 μm staurosporine (JHUEM2; Fig. 2C). We observed activation of caspase‐3, and furthermore, cell death resulting from these compounds was blocked by pretreatment with Z‐VAD‐FMK, indicating that classical apoptotic signalling is functional in these cells (Fig. 2D).

We went on to investigate the known mediators of CICD: AIF, EndoG, Smac/diablo and HTRA2/OMI. No translocation of Smac/diablo or HTRA2/OMI from the mitochondria to the cytosol was observed (Fig. 2E). We note that while the mitochondrial fraction also contains ER proteins, no loss of expression in the mitochondria fraction was observed nor any increase expression in the cytosol, suggesting no translocation from the mitochondria has taken place. Goke *et al*. (Goke *et al*., 2015a) reported an increase in AIF expression in the cytosol of the H1581 lung cancer cell line after 48‐h BGJ398 treatment and concluded that caspase‐independent apoptosis was occurring. Conversely, in our EC cell lines, translocation of AIF from the mitochondria to the nucleus was not observed (Fig. 2F) and shRNA‐mediated knockdown of AIF and EndoG failed to block FGFR inhibitor‐mediated cell death in AN3CA and JHUEM2 cells (Fig. S1A–H). We ruled out necroptosis, another form of CICD, by pretreating cells with 100 μm necrostatin, an inhibitor of the death domain receptor‐associated adaptor kinase RIP (RIP1). Necrostatin did not prohibit cell death by FGFR inhibitors in AN3CA or JHUEM2 cells (Fig. S1I,J). Furthermore, there was no upregulation in GRP78, a marker of endoplasmic reticulum stress, in AN3CA or JHUEM2 cells following BGJ398 treatment over 72 h (Fig. 4A), indicating the unfolded protein response was not induced by FGFR inhibition.

**Figure 3:**
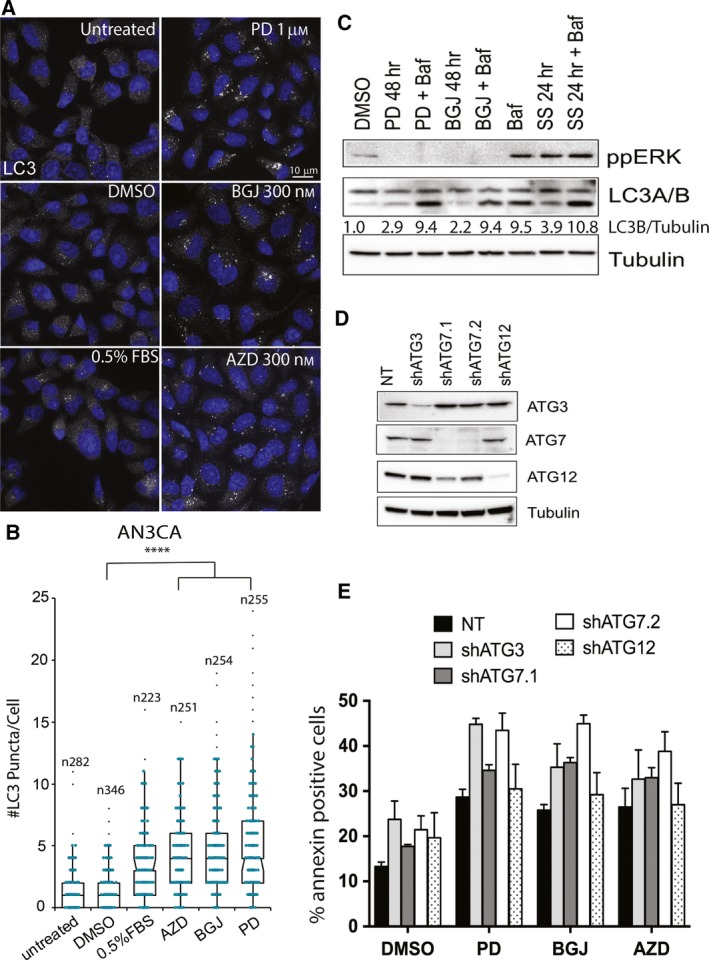
Fibroblast growth factor receptor inhibition increases autophagosome number in FGFR2‐mutant EC cells. **(**A) AN3CA cells untreated, serum‐starved in 0.5% FBS overnight or treated with the indicated concentrations of PD173074 (PD), BGJ398 (BGJ) and AZD4547 (AZD) for 48 h prior to staining for LC3. Scale bar represents 10 μm. (B) Quantitation of LC3 puncta per cell. 0.5% FBS was included as a positive control for autophagy induction. Ten fields were imaged for each condition using a 60x objective. *n* = # of puncta per cell. Error bars indicate SD. Data were analysed using a Kruskal–Wallis one‐way ANOVA with a Dunn's multiple comparison test. *P *<0.0001 (****). (C) Western blot of JHUEM2 cells treated with DMSO, 1 μm PD or 300 nm BGJ for 48 h with or without 20 nmol bafilomycin (Baf) for 4 h prior to collection. Cells grown in 0.5% FBS for 24 h (SS) −/+ Baf served as a positive control of autophagy induction. Phospho‐ERK1/2, LC3‐I (upper band) and LC3‐II (lower band) are shown along with the loading control tubulin. (D) Western blot of AN3CA cells stably expressing nontargeting shRNA control (NT) and shRNAs targeting ATG3, ATG7 and ATG12. (E) Percentage of AN3CA cells staining positive for Annexin V following treatment with PD, BGJ or AZD for 72 h. Error bars indicate SD.

Autophagy, the process of intracellular self‐digestion in autophagosomes and autolysosomes, is another form of programmed cell death we investigated. We observed a significant increase in the number of LC3 (autophagosomal marker) puncta per cell (*P *<* *0.0001) following the treatment of AN3CA and JHUEM2 cells with PD173074, BGJ398 and AZD4547 (Figs 3A,B and S2A,B). In order to distinguish whether the increase in LC3 puncta was due to increased autophagosome synthesis or a block in autolysosomal fusion/degradation, we sort to assess the level of lipidated LC3‐II in the presence and absence of bafilomycin. Starving the cells in 0.5% FBS overnight served as a positive control for autophagy induction. Bafilomycin, an inhibitor of LC3‐II degradation, caused accumulation of LC3‐II, with the combination of starvation and bafilomycin leading to greater accumulation of LC3‐II. In contrast, although FGFR inhibition induced an increase in lipidated LC3‐II, bafilomycin did not increase the levels of LC3‐II more than bafilomycin alone (Fig. 3C), suggesting the increase in LC3 puncta is due to a block in autophagosome fusion/degradation rather than an increase in autophagosome synthesis.

When we depleted the key autophagy proteins ATG3, 7 and 12 via shRNA knockdown, there was no reduction in cell death by FGFR inhibitors (Fig. 3D,E), indicating that the effect of FGFR inhibition on autophagic flux is not involved in the cell death observed. Autophagy has been paradoxically implicated in both cell death and cell survival by providing an alternative energy source and maintaining cell homoeostasis (Kourtis and Tavernarakis, 2009). These results suggest that autophagy is not contributing to cell destruction; however, further work is required to investigate the effect of FGFR inhibitors on autolysosomal fusion and lysosomal degradation.

**Figure 4:**
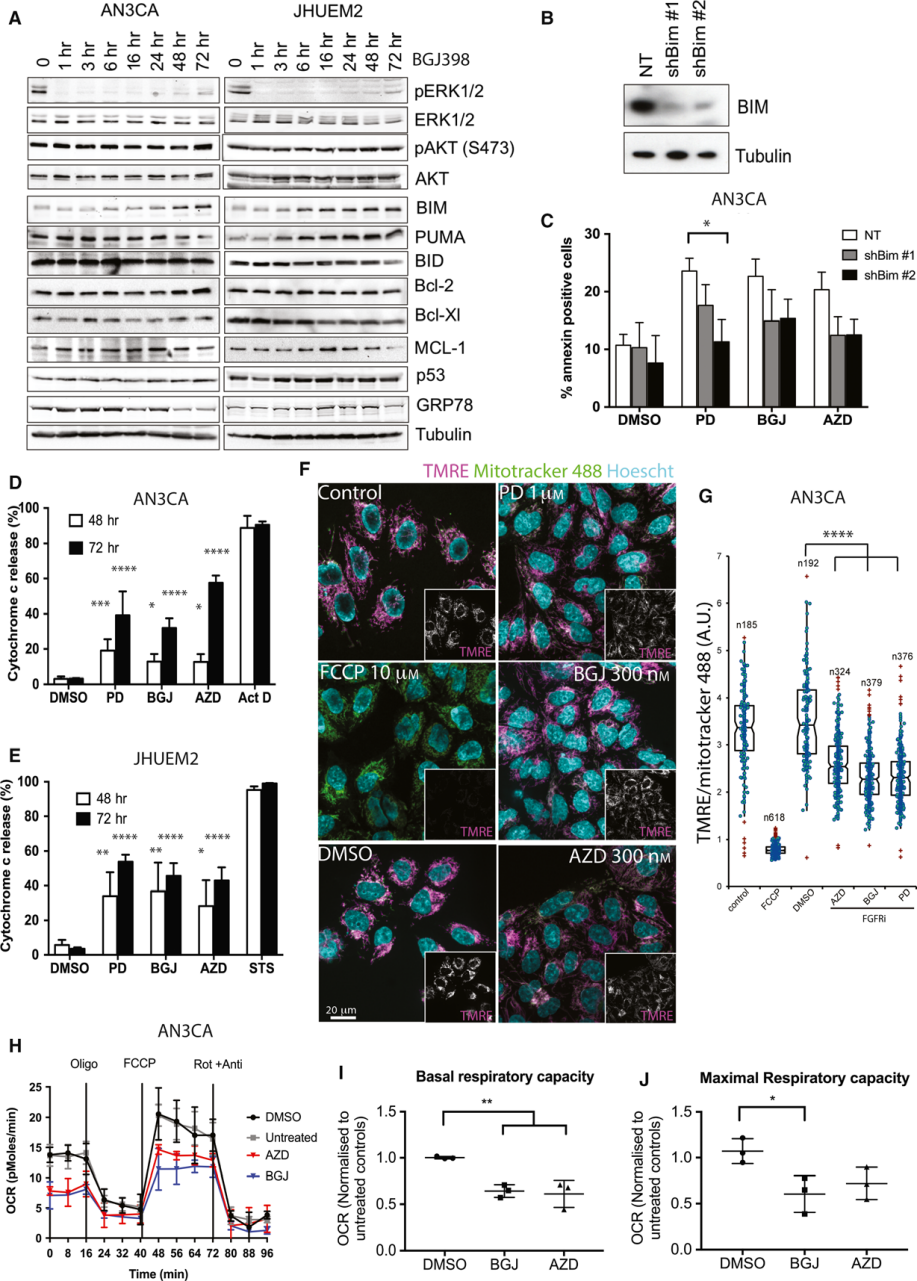
Cell death induced by FGFR inhibition involves mitochondrial dysfunction. (A) Western blot showing AN3CA and JHUEM2 cells treated with 300 nm BGJ398 for up to 72 h. Various effectors of FGFR signalling and cell death are shown, with tubulin as the loading control. (B) Western blot of AN3CA cells stably expressing nontargeting shRNA control (NT) and two different Bim shRNAs. (C) Percentage of AN3CA cells (expressing NT or Bim shRNAs) staining positive for Annexin V following 72‐h treatment with DMSO, 1 μm PD173074 (PD), 300 nm BGJ398 (BGJ) or 300 nm AZD4547 (AZD), along with SD. Percentage of AN3CA (D) and JHUEM2 (E) cells showing cytochrome c release following 48‐ or 74‐h treatment with DMSO, PD, BGJ or AZD. 1 μm actinomycin D (Act D; AN3CA) and staurosporine (STS; JHUEM2) were used as positive controls of cytochrome c release. Error bars indicate SD. Data from C‐E were analysed using a one‐way ANOVA with Dunnett's multiple comparison to compare treatments to control. (F) AN3CA cells untreated (control) or treated with DMSO or the indicated concentrations of PD, BGJ or AZD for 48 h prior to staining with MitoTracker Green FM and TMRE and imaged live on a spinning disc microscope. Treatment of cells with 10 μm FCCP for 10 min served as a control of mitochondrial membrane depolarization. Scale bar represents 20 μm. (G) Quantification of TMRE staining in AN3CA cells shown in F). TMRE staining is normalized to MitoTracker staining (to indicate total mitochondrial mass) per cell. *N* = number of cells counted in a single experiment representative of four independent experiments. Error bars show SD. Data were analysed using a Kruskal–Wallis one‐way ANOVA with a Dunn's multiple comparison test. (H) Mitochondrial respiration reflected by oxygen consumption rate (pmoles·min^−1^) was detected in AN3CA cells untreated (grey line), or treated with DMSO (black line), 300 nm BGJ (blue line) or 300 nm AZD (red line) for 48 h under basal conditions or following the addition of 1.2 μm oligomycin, the uncoupler FCCP (1 μm) or the electron transport inhibitor rotenone and antimycin (each at 1 μm), along with the SD. Basal (I) and maximal (J) respiratory capacity were quantified by normalization of OCR level to the total cell number and then expressed as a ratio of the untreated control. Error bars show SD. Results of a one‐way ANOVA with Dunnett's multiple comparison are shown. *P <* *0.05, **P <* *0.01, ***P <* *0.001, ****P <* *0.0001.

We further investigated the role of mitochondria in cell death resulting from FGFR inhibition. Markers of mitochondrial cell death were measured by western blotting over 72 h of BGJ398 treatment (Figs 4A and S3A,B). Although MAPK/ERK signalling has been shown to regulate the transcription of prosurvival Bcl‐2 proteins in different cellular systems (Boucher *et al*., 2000; Jost *et al*., 2001), no change in expression was seen in Bax, Bcl‐2, Bcl‐XL or Mcl‐1 in AN3CA or JHUEM2 cells over 72 h despite robust ERK inhibition (Fig. 4A and data not shown). PUMA was upregulated in JHUEM2 (which harbours wild‐type p53); however, no such change was observed in AN3CA (which harbours mutant p53^R213Q^). No change was observed in Bax, Bid, Mcl‐1 or p53 (Fig. 4A and data not shown). In line with its role as an important target of MAPK/ERK signalling, there was a marked upregulation of the BH3‐only protein Bim in AN3CA and JHUEM2 cells 6–16 h after BGJ398 treatment. ERK phosphorylates Bim, leading to degradation by the proteasome pathway (Luciano *et al*., 2003). The downward shift in Bim observed with BGJ398 treatment (Fig. 4A) is likely due to Bim dephosphorylation downstream of ERK inhibition, resulting in Bim accumulation and enabling Bim binding to Bcl‐2 family members. This is considered to be an initiating event in the cell death cascade (Puthalakath *et al*., 1999). Depletion of Bim by shRNA abrogated cell death in AN3CA cells (Fig. 4B,C), confirming the role of Bim in FGFR inhibitor‐mediated cell death.

Induction of BH3‐only proteins neutralizes the prosurvival proteins such as Bcl‐2, Bcl‐XL and Mcl‐1, enabling activation of Bax and Bak on the mitochondrial outer membrane, which is accompanied by cytochrome c release and membrane permeabilization. We investigated the effect of FGFR inhibitors on cytochrome c release from the mitochondria (Fig. 4D,E). Cells were treated with PD173074, BGJ398 and AZD4547 for 48 and 72 h, after which cells were digitonin‐permeabilized, fixed, stained with cytochrome c and assessed by flow cytometry. Both AN3CA and JHUEM2 cells showed a significant increase in cytochrome c release from the mitochondria following FGFR inhibition. AN3CA cells showed a ~12% (*P *<* *0.05) increase in cytochrome c release at 48 h and a > 28% (*P *<* *0.0001) increase following 72 h of FGFR inhibition. Cytochrome c release in JHUEM2 cells increased ~25% (*P *<* *0.05) at 48 h and ~40% (*P *<* *0.0001) at 72 h. As a positive control of apoptosis, cells were treated with 1 μm actinomycin D (AN3CA) or staurosporine (JHUEM2).

Release of cytochrome c from the mitochondria is typically linked with perturbation of mitochondrial function, via mitochondrial outer membrane permeabilization (MOMP). To determine whether FGFR inhibition in FGFR2‐mutant EC cells caused depolarization of the mitochondrial membrane, we stained live cells with TMRE and assessed by spinning disc confocal microscopy 48 h post‐treatment (Figs 4F,G and S3C,D). FCCP, a mitochondrial uncoupler, caused complete loss of TMRE staining. TMRE staining was normalized to total mitochondrial staining by MitoTracker Green FM to account for mitochondrial mass. Our results show that mitochondrial membrane potential was significantly reduced (*P *<* *0.0001) in AN3CA and JHUEM2 cells treated with FGFR inhibitors (Figs 4G and S3D).

To further investigate the demise of mitochondrial function and bioenergetics, we used the Seahorse XF24 assay to measure mitochondrial respiration (oxygen consumption) and glycolysis (extracellular acidification). We observed no change in the extracellular acidification rate (ECAR) (data not shown), a measurement of glycolytic function, in AN3CA or JHUEM2 cells at 24 and 48 h of treatment with FGFR inhibitors. To measure the oxygen consumption rate (OCR) of cells, sequential injection of compounds that target the electron transport chain such as oligomycin (oligo), FCCP and rotenone and antimycin A (Rot + Anti) was performed to measure ATP production, maximal respiration and nonmitochondrial respiration (Fig. 4H). Basal respiration and spare respiratory capacity are then calculated using these parameters. A significant reduction in basal ADP‐stimulated respiration and maximal mitochondrial respiratory capacity was observed in AN3CA cells treated with FGFR inhibitors BGJ398 and AZD4547 for 48 h (Fig. 4H–J), indicating that there was a marked reduction in oxidative phosphorylation capacity in cells treated with FGFR inhibitors.

**Figure 5:**
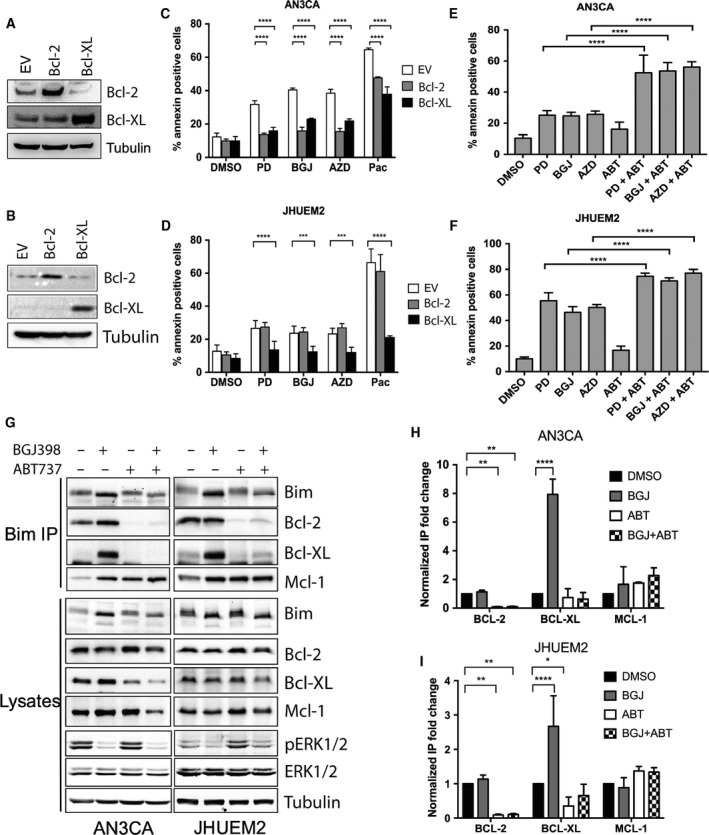
Bcl‐2 inhibitors augment cell death induced by FGFR inhibitors. Western blot showing AN3CA (A) and JHUEM2 (B) cells stably expressing the empty vector control (EV), Bcl‐2 or Bcl‐XL. Percentage of Annexin V‐positive AN3CA (C) and JHUEM2 (D) cells expressing EV, Bcl‐2 or Bcl‐XL treated with DMSO, 1 μm PD173074 (PD), 300 nm BGJ398 (BGJ), 300 nm AZD4547 (AZD) or 10 nm paclitaxel (Pac) for 72 h. Percentage of AN3CA (E) and JHUEM2 (F) cells positive for Annexin V following 72‐h treatment with DMSO and the above concentrations of PD, BGJ and AZD and 1 μm ABT737 alone or in combination. Error bars show SD. Comparison of treatment groups to DMSO control in C–F was performed using one‐way ANOVA with Dunnett's multiple comparison. (G) Western blotting showing Bim, Bcl‐2, Bcl‐XL, Mcl‐1 and tubulin (loading control) in AN3CA and JHUEM2 cell lysates or Bim co‐IPs 24 h after treatment with 300 nm BGJ and/or 1 μm ABT737. Graphs (H) and (I) show quantification of Bim co‐IPs (normalized to total Bim) from triplicate experiments shown in (G). Error bars indicate SD. A two‐way ANOVA with Holm–Sidak multiple comparisons correction tests was used to analyse the data in H‐I. *P <* *0.05, **P <* *0.01, ***P <* *0.001, ****P <* *0.0001.

We then confirmed mitochondrial cell death by overexpression of Bcl‐2 and Bcl‐XL, which impede cell death by inhibiting both the BH3 proteins and the Bax/Bak executioner proteins (Fig. 5A–D). In AN3CA cells, overexpression of both Bcl‐2 and Bcl‐XL prevented cell death by the FGFR inhibitors (*P *<* *0.0001). In contrast, only overexpression of Bcl‐XL blocked cell death by PD173074, BGJ398 and AZD4547 (*P *<* *0.001) in JHUEM2 cells. This is consistent with the induction of Bim:Bcl‐XL but not Bim:Bcl‐2 complex formation by BGJ398 in these cells (Fig. 5G–I). These results suggest that JHUEM2 relies more on Bcl‐XL than Bcl‐2 for maintaining mitochondrial integrity.

Our data suggest that the antitumour effect of FGFR inhibitors could be enhanced by BH3 mimetics such as ABT737, which binds with high affinity to Bcl‐2 and Bcl‐XL. While 1 μm of ABT737 did not significantly increase cell death alone, when used in combination with FGFR inhibitor ABT737 we observed a significant increase in cell death in AN3CA, JHUEM2 and an additional FGFR2‐mutant EC cell line MFE296, compared to that induced by FGFR inhibitors alone (*P *<* *0001; Figs 5E,F and S4B). To confirm the inhibition of Bcl‐2/XL by ABT737, Bim was immunoprecipitated and probed for Bcl‐2, Bcl‐XL and Mcl‐1 (Figs 5G and S4C). Bim appeared to be predominantly bound to Bcl‐2 and Mcl‐1 under normal conditions. While FGFR inhibition did not affect Bim binding to Bcl‐2 or Mcl‐1 (relative to Bim levels), it significantly increased Bim binding to Bcl‐XL in AN3CA, JHUEM2 and MFE296 (*P *<* *0.0001; Figs 5G–I and S4D). ABT737 inhibited binding of Bim to Bcl‐2 and blocked Bcl‐XL binding to Bim in the presence of BGJ398. Thus, ABT737 potentiates the displacement of Bcl‐2 and Bcl‐XL from BH3 activators such as Bim and also from the apoptotic executioners Bax and Bak on the mitochondrial membrane, to enhance mitochondrial‐dependent cell death following FGFR inhibition by BGJ398.

**Figure 6:**
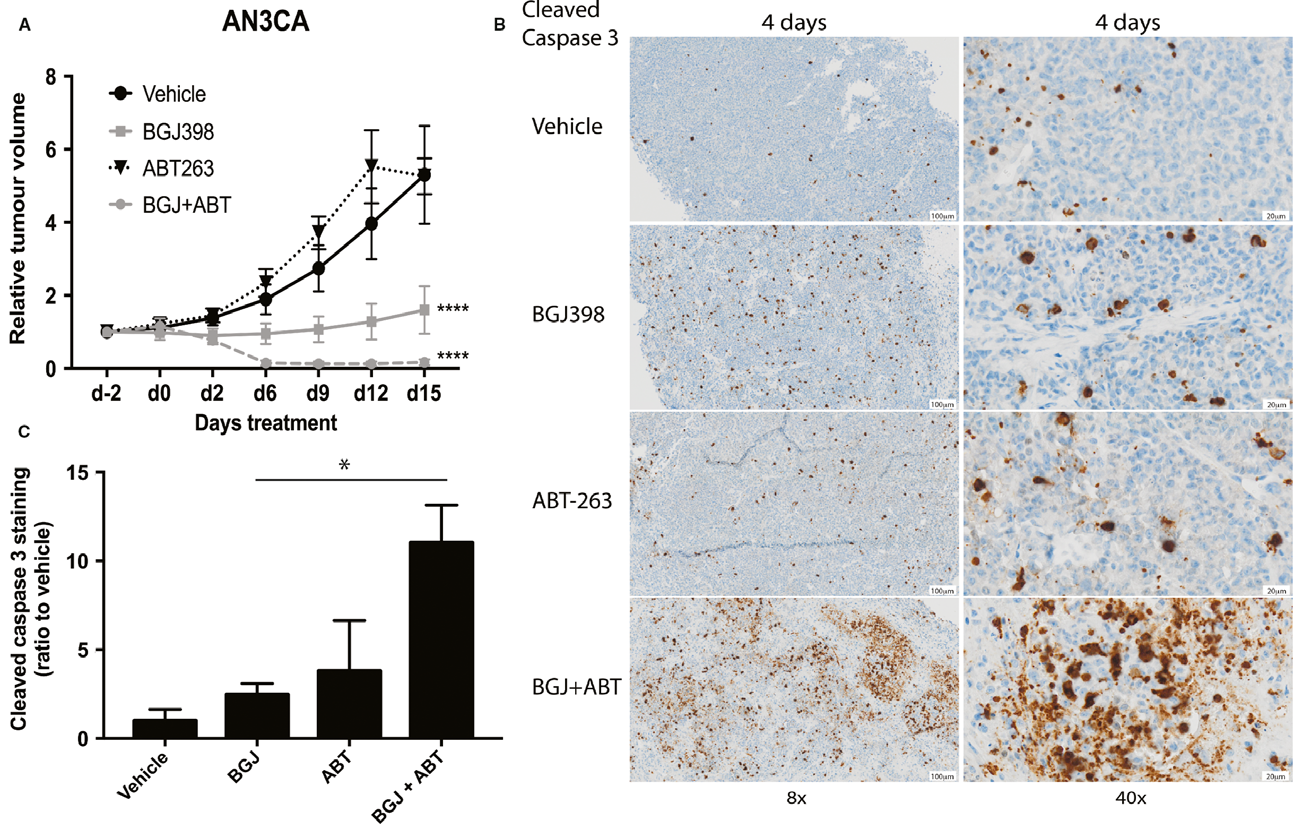
Bcl‐2 inhibitor ABT263 and BGJ398 cooperate to kill FGFR2‐mutant AN3CA cells *in vivo*. (A) AN3CA xenografts were established in NSG mice and stratified into 4 groups (8/group) then treated for 2 weeks via oral gavage (6 days on, 1 day off) with vehicle, 20 mg·kg^−1^ BGJ398 or 100 mg·kg^−1^ ABT263, or BGJ398 +  ABT263. Mean tumour volumes are shown along with SEM. Differences in xenograft volume between groups were assessed for significance using a repeated two‐way ANOVA with Tukey's multiple comparisons test. (B) Immunohistochemical staining of cleaved caspase‐3 in AN3CA xenografts treated for 4 days with the indicated drugs. Scale bar represents 100 μm (8x objective) and 20 μm (40x objective). (C) Mean of cleaved caspase‐3‐positive cells as quantified by visiopharm integrator system v2107.2 (presented as a ratio over vehicle control). Error bars show SD. A one‐way ANOVA with Tukey's multiple correction test was used to analyse the data. *P <* *0.05, ****P <* *0.0001.

We further investigated the combination of Bcl‐2 inhibitor and FGFR inhibitor *in vivo* with AN3CA cells grown as xenografts in NSG mice. ABT737 is not orally bioavailable, so we used its orally active analogue ABT263 (navitoclax). We treated mice by oral gavage once daily with BGJ398 (20 mg·kg^−1^) or ABT263 (100 mg·kg^−1^) alone or in combination for 15 days. Tumour growth is shown in Fig. 6A. When used in combination with BGJ398, ABT263 caused marked tumour regression. Overall, the combination of BGJ398 +  ABT263 significantly improved the antitumour response to BGJ398 alone (*P *<* *0.01). Assessment of caspase cleavage in AN3CA xenografts following 4 days of treatment revealed an increase in caspase cleavage with BGJ398 (2.5‐fold) or ABT263 (3.8‐fold) given as single agents compared to vehicle control (Fig. 6B,C). The combination of BGJ398 +  ABT263 caused an 11‐fold increase in caspase‐3 cleavage compared to the control (*P* < 0.05). These results suggest that BGJ398 +  ABT263 enhances tumour cell death by caspase cleavage and activation of the classical apoptotic pathway.

## 4 Discussion

We previously reported cell death, evidenced by increased annexin positivity, in FGFR2‐mutated EC cell lines treated with PD173074 (Byron *et al*., 2008). Annexin‐positive cell death was subsequently reported 72 h after treatment with BGJ398 (Konecny *et al*., 2015), and following 48‐h treatment of AN3CA and MFE296 by AZD4547 (Kwak *et al*., 2015). No cell death, evidenced by lack of a subG1 peak, was reported in AN3CA cells following 24‐h treatment with ponatinib, ridaforolimus (mTOR inhibitor) and their combination (Gozgit *et al*., 2013), thus highlighting the need to assess cell death at multiple time points. As the cell death we observed in FGFR2‐dependent EC cell lines following FGFR inhibition is unique, in that it occurs in the presence of constitutive PI3K activity, we sought to explore the molecular events underpinning this cell death in detail.

We have shown that FGFR inhibition and subsequent inhibition of the MAPK pathway in FGFR2‐mutant EC result in Bim upregulation, as has been previously reported (Gillings *et al*., 2009). Bim stabilization has been shown to sequester the prosurvival proteins Bcl‐2 and Bcl‐XL, which ultimately leads to impairment of mitochondrial function, through cytochrome c release and MOMP. Frameshift mutations in Bax, leading to loss of expression, which in turn impede apoptosis through an increased Bcl‐2/Bax ratio, were previously identified in ~10% of endometrial cancers (Sakuragi *et al*., 2002). Both AN3CA and MFE296 contain heterozygous frameshift mutations in Bax (p.A184 fs*29 and p.M38 fs, respectively). However, western blot analysis showed that full‐length wild‐type Bax protein is expressed in these cells (Fig. S4A).

Our results also indicated that FGFR inhibition reduced the basal and maximal respiratory capacity of the mitochondria (Fig. 4H–J), placing the cells in a state of energy stress. However, without a concurrent increase in glycolysis (ECAR), we propose this energy stress ultimately leads to delayed cell death. Interestingly, we did not detect caspase cleavage via western blotting over a 72‐h time course of FGFR inhibition in either AN3CA or JHUEM2 (Fig. 2A) and pretreatment with a pan‐caspase inhibitor was unable to block cell death following FGFR inhibition (Fig. 2B), despite the capacity for caspase‐dependent cell death in these cells (Fig. 2C,D). Significant cell death in the absence of caspase‐3/7 activity has been reported in several other FGFR‐dependent models including breast cancer (CAL51) (Sharpe *et al*., 2011) and mesothelioma (H2810) (Quispel‐Janssen *et al*., 2018). Although caspase and/or PARP cleavage following FGFR inhibition has been reported in several gastric and breast models (Kunii *et al*., 2008; Pearson *et al*., 2016), it has only been reported in one liver cell line (Hep3B) (Hagel *et al*., 2015) and one lung cancer cell line (H1581) (Goke *et al*., 2015a) with caspase‐independent death also being reported in the latter. In addition, cell death following FGFR inhibition with PD173074 in bladder cancer models has only been identified based on annexin positivity (Lamont *et al*., 2011); hence, this caspase‐independent cell death could be present in other FGFR‐dependent cancer types.

Our *in vivo* studies showed ~3% of AN3CA cells grown as xenografts stained positive for cleaved caspase‐3 (Fig. 6B) following BGJ398 treatment, compared to ~1% in vehicle‐treated controls, while caspase activation was significantly increased when BGJ398 was combined with ABT263. Whether the caspase cleavage in xenografts treated with BGJ398 alone indicates a low level of caspase cleavage undetectable by western blot analysis, or alternatively whether caspase‐dependent death is due to *in vivo* hypoxia, is unknown. Nevertheless, the combination of Bcl‐2 inhibition by ABT263 and Bim upregulation by BGJ398 triggers substantial caspase activation in the tumour, which likely contributes to the enhanced cell death following treatment with BGJ398 +  ABT263.

Autophagy has been previously reported in FGFR1‐amplified lung cancer models and a single breast cancer line following FGFR inhibition with AZD4547 (Yuan *et al*., 2017) or PD166866, respectively (Chen *et al*., 2016). In contrast to our findings in EC, FGFR inhibition in combination with either lysosomal protease inhibitors or chloroquine leads to a significant increase in lipidated LC3‐II or LC3 puncta in these lines, suggesting that FGFR inhibition increases autophagosome synthesis. Both Yuan *et al*. (2017) and Chen *et al*. (2016) found that blocking autophagy by knockdown of Beclin and ATG5 increased cell death by FGFR inhibition. Further studies of FGFR inhibition on autophagic flux in the context of activation of different FGFRs are required.

We have now shown that FGFR inhibition results in inhibition of the MAPK pathway and the induction of Bim (Figs 2E, 4A) and that knockdown of Bim or overexpression of Bcl‐XL could block BGJ398‐ or AZD4547‐induced cell death (Figs 4C and 5A–D). BGJ398 treatment induced significantly more binding of Bim to Bcl‐XL (Fig. 5G) whereas no increase in the binding of Bim to Bcl‐2 was observed when change in immunoprecipitated Bcl‐2 (treated vs DMSO) was normalized to the change in total levels of Bim (Fig. 5G). As expected, cotreatment of cells with BGJ398 and the Bcl‐2/Xl inhibitor ABT737 prevented Bim:Bcl‐XL binding and led to a significant increase in annexin positivity *in vitro* (Fig. 5E,F). We confirmed this *in vivo* and report for the first time that the combination of BGJ398 and ABT263 treatment of AN3CA xenografts led to significant tumour regression (Fig. 6A). Although these cells do express Mcl‐1, we hypothesize that combining ABT263 with BGJ398 leads to a displacement of Bim from Bcl‐XL to Mcl‐1 leading to the effective induction of cell death. Very little is known about the relative role of Bcl‐2/Bcl‐XL/Mcl‐1 in other solid malignancies with FGFR1‐3 activation. Recently, a study in lung cancer cell lines and PDX models showed that combining BGJ398 with either the Bcl‐XL inhibitor A1331852 or the Mcl‐1 inhibitor S63845 led to a small survival benefit and that only triple inhibition resulted in tumour regression (Weeden *et al*., 2018). The reason for the disparate results between lung and endometrial cancer models is unknown but could be due to higher expression of Mcl‐1 in lung cancers and/or differences in other prosurvival/pro‐apoptotic proteins regulated by FGFR1 and FGFR2. The work presented here supports the evaluation of ABT263 or other Bcl‐XL inhibitors in combination with FGFR inhibition in other FGFR2‐ and FGFR3‐dependent cancers.

Studies have shown that cancers with high expression of FGFR mRNA and protein are more likely to respond to FGFR inhibitors (Goke *et al*., 2015b; Pearson *et al*., 2016; Weiss *et al*., 2010). In addition, we and others have shown that in preclinical models where FGFR inhibition does not block PI3K pathway activation, the combination of FGFR and AKT/PI3K/mTOR inhibition resulted in increased cell death (Fumarola *et al*., 2017; Packer *et al*., 2017; Yu *et al*., 2017). However, despite preclinical efficacy, dual FGFR/PI3K inhibition is unlikely to be evaluated in clinical trials due to its combined toxicity (Hyman *et al*., 2016). The current study has revealed that similar synergy can be obtained by targeting downstream prosurvival proteins following mitochondrial priming by FGFR inhibition.

Dual Bcl‐2 and Bcl‐XL inhibition with ABT263 (navitoclax) has shown single‐agent activity in a variety of leukaemias and lymphomas (Tse *et al*., 2008). Clinical testing of ABT263 was initially associated with thrombocytopenia in patients, due to the reliance of platelets on Bcl‐XL for survival (Mason *et al*., 2007), but changes in ABT263 dosing to include an initial ramping period have overcome the acute thrombocytopenia (Roberts *et al*., 2015). Consequently, ABT263 is again being tested in solid malignancies in combination with EGFR inhibition (NCT02520778), MEK inhibition (NCT02079740) or mTOR inhibition (NCT03366103). Our results provide evidence to suggest that in FGFR2‐mutant EC, Bim:Bcl‐XL binding is critical for mediating cell death downstream of FGFR inhibition. Thus, a Bcl‐XL inhibitor (e.g. A1331852) or dual Bcl‐2/Xl inhibitor (such as ABT263) should be tested with FGFR inhibitors in other FGFR‐dependent tumour models in order to determine the feasibility of a basket trial testing this combination in multiple FGFR‐dependent malignancies.

## 5 Conclusions

In this study, we show that FGFR inhibition in FGFR2‐mutant EC induces mitochondrial‐dependent cell death which involves induction of Bim, MOMP and cytochrome c release. Low‐level caspase activation is seen *in vivo* with BGJ398 treatment, which was significantly enhanced by ABT263, resulting in enhanced cell death and tumour regression *in vivo*. Our data indicate that priming the cell for apoptosis using BH3 mimetics could improve the clinical response to FGFR inhibitors in FGFR‐driven cancers, which have had disappointing responses to FGFR inhibitors given as single agents.

## Conflict of interest

Pollock is an inventor of two patents involving the detection of FGFR2 mutations for diagnostic or prognostic purposes in endometrial cancer. The remaining authors declare no conflicts of interests.

## Author contributions

LMP, SJS, VFB, SAB and PMP conceived and designed experiments. LMP, SJS, VFB, JHG, RJJ, MW, MGG and SAB conducted the experiments. LMP, SJS, VFB, JHG, MW, SAB and PMP contributed to interpretation of the data. LMP and PMP wrote the manuscript.

## Supporting information


**Fig. S1.** Western blots showing EndoG in AN3CA (A) and JHUEM2 (C) cells stably expressing nontargeting shRNA (NT), shEndoG 1 or shEndoG 2. AN3CA (B) and JHUEM2 (D) cells stably expressing NT shRNA, shEndoG 1 or shEndoG 2 were treated with 1 μm PD173074 (PD), 300 nm BGJ398 (BGJ), 300 nm AZD4547 (AZD) or 10 nm paclitaxel (Pac) for 72 h. Cell death was detected by staining cells with Annexin V. The mean percentage of Annexin V‐positive cells from three independent experiments (each performed in triplicate) is shown along with SD. Western blot showing levels of AIF in AN3CA (E) and JHUEM2 (G) cells stably expressing NT shRNA, shAIF #1 and shAIF #2. AN3CA (F) and JHUEM2 (H) cells stably expressing NT shRNA, shAIF #1 and shAIF #2 were treated with the above concentrations of PD, BGJ and AZD for 72 h. Cell death was detected by staining cells with Annexin V. The mean percentage of Annexin V‐positive cells from three independent experiments (each performed in triplicate) is shown along with SD. AN3CA (I) and JHUEM2 (J) cells were treated with 1 μm PD, 300 nm BGJ +/− 100 μm necrostatin for 72 h. Cell death was detected by staining cells with Annexin V. The mean percentage of Annexin V‐positive cells from three independent experiments (each performed in triplicate) is shown along with SD. *P* < 0.05 (*), <0.01 (**), <0.001 (***), <0.0001 (****).

**Fig. S2.** (A) JHUEM2 cells untreated, serum‐starved in 0.5% FBS overnight or treated with the indicated concentrations of PD173074 (PD), BGJ398 (BGJ) and AZD4547 (AZD) for 48 h prior to staining for LC3. (B) Quantitation of LC3 puncta per cell. 0.5% FBS was included as a positive control for autophagy induction. 10 fields were imaged for each condition using a 60x objective. *n* = per cell. Error bars show SD. Kruskal–Wallis one‐way ANOVA with a Dunn's multiple comparison test. *P *<* *0.05 (*), <0.01 (**), <0.001 (***), <0.0001 (****).

**Fig. S3.** Densitometric analysis of AN3CA (A) and JHUEM2 (B) western blots shown in Figure 4A performed in biological triplicate. Proteins were normalized to tubulin and then expressed as a fold change of the DMSO control, with the exception of phospho‐ERK1/2 and phospho‐AKT, which were normalized to total ERK1/2 and total AKT, respectively. Error bars indicate SD. (C) JHUEM2 cells untreated (control) or treated with DMSO or the indicated concentrations of PD173074 (PD), BGJ398 (BGJ) or AZD4547 (AZD) for 48 h prior to being stained with MitoTracker Green FM and TMRE and imaged on a spinning disc microscope. Treatment of cells with 10 μm FCCP for 10 min served as a control of mitochondrial membrane depolarization. (D) Quantification of TMRE staining in JHUEM2 cells shown in (C) using a Kruskal–Wallis one‐way ANOVA with a Dunn's multiple comparison test. TMRE staining is normalized to MitoTracker staining (to indicate total mitochondrial mass) per cell. *N* = number of cells counted. *P *<* *0.05 (*), <0.01 (**), <0.001 (***), <0.0001 (****).

**Fig. S4.** (A) Western blot showing AN3CA, JHUEM2 and MFE296 cells treated with 1 μm PD173074 for up to 72 h. Bim and Bax levels are shown, with tubulin as the loading control. (B) Percentage of MFE296 cells positive for Annexin V following 72‐h treatment with DMSO, 1 μm PD173074 (PD), 300 nm BGJ398 (BGJ), 300 nm AZD4547 (AZD) and 1 μm ABT737 alone or in combination. One‐way ANOVA with Dunnett's multiple comparison to compare treatments to control. Error bars show SD. *P *<* *0.05 (*), <0.01 (**), <0.001 (***), <0.0001 (****). (C) Western blotting showing Bim, Bcl‐2, Bcl‐XL, Mcl‐1 and tubulin (loading control) in AN3CA and JHUEM2 cell lysates or Bim co‐IPs 24 h after treatment with BGJ and/or ABT737. (D) Graph showing normalized quantification of Bim co‐IPs from triplicate experiments for samples shown in (B). Error bars show SD.

## References

[mol212422-bib-0001] Babina IS and Turner NC (2017) Advances and challenges in targeting FGFR signalling in cancer. Nat Rev Cancer 17, 318–332. 28303906 10.1038/nrc.2017.8

[mol212422-bib-0002] Boucher MJ , Morisset J , Vachon PH , Reed JC , Laine J and Rivard N (2000) MEK/ERK signaling pathway regulates the expression of Bcl‐2, Bcl‐X(L), and Mcl‐1 and promotes survival of human pancreatic cancer cells. J Cell Biochem 79, 355–369. 10972974

[mol212422-bib-0003] Brand MD and Nicholls DG (2011) Assessing mitochondrial dysfunction in cells. Biochem J 435, 297–312. 21726199 10.1042/BJ20110162 PMC3076726

[mol212422-bib-0004] Byron SA , Gartside M , Powell MA , Wellens CL , Gao F , Mutch DG , Goodfellow PJ , Pollock PM (2012) FGFR2 point mutations in 466 endometrioid endometrial tumors: relationship with MSI, KRAS, PIK3CA, CTNNB1 mutations and clinicopathological features. PLoS ONE 7, e30801. 22383975 10.1371/journal.pone.0030801 PMC3285611

[mol212422-bib-0005] Byron SA , Gartside MG , Wellens CL , Mallon MA , Keenan JB , Powell MA , Goodfellow PJ , Pollock PM (2008) Inhibition of activated fibroblast growth factor receptor 2 in endometrial cancer cells induces cell death despite PTEN abrogation. Can Res 68, 6902–6907. 10.1158/0008-5472.CAN-08-0770 18757403

[mol212422-bib-0006] Chell V , Balmanno K , Little AS , Wilson M , Andrews S , Blockley L , Hampson M , Gavine PR , Cook SJ (2013) Tumour cell responses to new fibroblast growth factor receptor tyrosine kinase inhibitors and identification of a gatekeeper mutation in FGFR3 as a mechanism of acquired resistance. Oncogene 32, 3059–3070. 22869148 10.1038/onc.2012.319

[mol212422-bib-0007] Chen Y , Xie X , Li X , Wang P , Jing Q , Yue J , Liu Y , Cheng Z , Li J , Song H *et al* (2016) FGFR antagonist induces protective autophagy in FGFR1‐amplified breast cancer cell. Biochem Biophys Res Comm 474, 1–7. 26993162 10.1016/j.bbrc.2016.03.017

[mol212422-bib-0008] Christensen ME , Jansen ES , Sanchez W , Waterhouse NJ (2013) Flow cytometry based assays for the measurement of apoptosis‐associated mitochondrial membrane depolarisation and cytochrome c release. Methods (San Diego, Calif) 61, 138–145. 10.1016/j.ymeth.2013.03.020 23545197

[mol212422-bib-0009] Fumarola C , Cretella D , La Monica S , Bonelli MA , Alfieri R , Caffarra C , Quaini F , Madeddu D , Falco A , Cavazzoni A *et al* (2017) Enhancement of the anti‐tumor activity of FGFR1 inhibition in squamous cell lung cancer by targeting downstream signaling involved in glucose metabolism. Oncotarget 8, 91841–91859. 29190880 10.18632/oncotarget.19279 PMC5696146

[mol212422-bib-0010] Gavine PR , Mooney L , Kilgour E , Thomas A , Al‐Kadhimi K , Beck S , Rooney C , Coleman T , Baker D , Mellor MJ *et al* (2012) AZD4547: an orally bioavailable, potent, and selective inhibitor of the fibroblast growth factor receptor tyrosine kinase family. Cancer Res 72, 2045–2056. 22369928 10.1158/0008-5472.CAN-11-3034

[mol212422-bib-0011] Gillings AS , Balmanno K , Wiggins CM , Johnson M and Cook SJ (2009) Apoptosis and autophagy: BIM as a mediator of tumour cell death in response to oncogene‐targeted therapeutics. FEBS J 276, 6050–6062. 19788418 10.1111/j.1742-4658.2009.07329.x

[mol212422-bib-0012] Goke F , Franzen A , Hinz TK , Marek LA , Yoon P , Sharma R , Bode M , von Maessenhausen A , Lankat‐Buttgereit B , Goke A *et al* (2015b) FGFR1 expression levels predict BGJ398 sensitivity of FGFR1‐dependent head and neck squamous cell cancers. Clin Cancer Res 21, 4356–4364. 26015511 10.1158/1078-0432.CCR-14-3357 PMC4592392

[mol212422-bib-0013] Goke A , Goke R , Ofner A , Herbst A and Lankat‐Buttgereit B (2015a) The FGFR inhibitor NVP‐BGJ398 induces NSCLC cell death by activating caspase‐dependent pathways as well as caspase‐independent apoptosis. Anticancer Res 35, 5873–5879. 26504010

[mol212422-bib-0014] Gozgit JM , Squillace RM , Wongchenko MJ , Miller D , Wardwell S , Mohemmad Q , Narasimhan NI , Wang F , Clackson T , Rivera VM (2013) Combined targeting of FGFR2 and mTOR by ponatinib and ridaforolimus results in synergistic antitumor activity in FGFR2 mutant endometrial cancer models. Cancer Chemother Pharmacol 71, 1315–1323. 23468082 10.1007/s00280-013-2131-z

[mol212422-bib-0015] Hagel M , Miduturu C , Sheets M , Rubin N , Weng W , Stransky N , Bifulco N , Kim JL , Hodous B , Brooijmans N *et al* (2015) First selective small molecule inhibitor of FGFR4 for the treatment of hepatocellular carcinomas with an activated FGFR4 signaling pathway. Cancer Discov 5, 424. 25776529 10.1158/2159-8290.CD-14-1029

[mol212422-bib-0016] Hall TG , Yu Y , Eathiraj S , Wang Y , Savage RE , Lapierre JM , Schwartz B , Abbadessa G (2016) Preclinical activity of ARQ 087, a novel inhibitor targeting FGFR dysregulation. PLoS One 11, e0162594. 27627808 10.1371/journal.pone.0162594 PMC5023172

[mol212422-bib-0017] Holden P and Horton WA (2009) Crude subcellular fractionation of cultured mammalian cell lines. BMC Res Notes 2, 243. 20003239 10.1186/1756-0500-2-243 PMC2802353

[mol212422-bib-0018] Hyman DM , Tran B , Corral Jaime J , Garralda E , Machiels J‐PH , Schellens JHM , Bedard P , Campone M , Cassier P , Sarantopoulos J *et al* (2016) Phase Ib study of BGJ398 in combination with BYL719 in patients (pts) with select advanced solid tumors. J Clin Oncol 34, 2500.

[mol212422-bib-0019] Jeske YW , Ali S , Byron SA , Gao F , Mannel RS , Ghebre RG , DiSilvestro PA , Lele SB , Pearl ML , Schmidt AP *et al* (2017) FGFR2 mutations are associated with poor outcomes in endometrioid endometrial cancer: An NRG Oncology/Gynecologic Oncology Group study. Gynecol Oncol 145, 366–373. 28314589 10.1016/j.ygyno.2017.02.031 PMC5433848

[mol212422-bib-0020] Jost M , Huggett TM , Kari C , Boise LH and Rodeck U (2001) Epidermal growth factor receptor‐dependent control of keratinocyte survival and Bcl‐xL expression through a MEK‐dependent pathway. J Biol Chem 276, 6320–6326. 11098053 10.1074/jbc.M008210200

[mol212422-bib-0021] Konecny GE , Finkler N , Garcia AA , Lorusso D , Lee PS , Rocconi RP , Fong PC , Squires M , Mishra K , Upalawanna A *et al* (2015) Second‐line dovitinib (TKI258) in patients with FGFR2‐mutated or FGFR2‐non‐mutated advanced or metastatic endometrial cancer: a non‐randomised, open‐label, two‐group, two‐stage, phase 2 study. Lancet Oncol 16, 686–694. 25981814 10.1016/S1470-2045(15)70159-2

[mol212422-bib-0022] Konecny GE , Kolarova T , O'Brien NA , Winterhoff B , Yang G , Qi J , Qi Z , Venkatesan N , Ayala R , Luo T *et al* (2013) Activity of the fibroblast growth factor receptor inhibitors dovitinib (TKI258) and NVP‐BGJ398 in human endometrial cancer cells. Mol Cancer Ther 12, 632–642. 23443805 10.1158/1535-7163.MCT-12-0999

[mol212422-bib-0023] Koneczny I , Schulenburg A , Hudec X , Knofler M , Holzmann K , Piazza G , Reynolds R , Valent P , Marian B (2015) Autocrine fibroblast growth factor 18 signaling mediates Wnt‐dependent stimulation of CD44‐positive human colorectal adenoma cells. Mol Carcinog 54, 789–799. 24619956 10.1002/mc.22146 PMC4162857

[mol212422-bib-0024] Kourtis N and Tavernarakis N (2009) Autophagy and cell death in model organisms. Cell Death Differ 16, 21–30. 19079286 10.1038/cdd.2008.120

[mol212422-bib-0025] Kunii K , Davis L , Gorenstein J , Hatch H , Yashiro M , Di Bacco A , Elbi C , Lutterbach B (2008) FGFR2‐amplified gastric cancer cell lines require FGFR2 and Erbb3 signaling for growth and survival. Can Res 68, 2340–2348. 10.1158/0008-5472.CAN-07-5229 18381441

[mol212422-bib-0026] Kwak Y , Cho H , Hur W and Sim T (2015) Antitumor effects and mechanisms of AZD4547 on FGFR2‐deregulated endometrial cancer cells. Mol Cancer Ther 14, 2292–2302. 26294741 10.1158/1535-7163.MCT-15-0032

[mol212422-bib-0027] Lamont FR , Tomlinson DC , Cooper PA , Shnyder SD , Chester JD and Knowles MA (2011) Small molecule FGF receptor inhibitors block FGFR‐dependent urothelial carcinoma growth in vitro and in vivo. Br J Cancer 104, 75–82. 21119661 10.1038/sj.bjc.6606016 PMC3039817

[mol212422-bib-0028] Luciano F , Jacquel A , Colosetti P , Herrant M , Cagnol S , Pages G and Auberger P (2003) Phosphorylation of Bim‐EL by Erk1/2 on serine 69 promotes its degradation via the proteasome pathway and regulates its proapoptotic function. Oncogene 22, 6785–6793. 14555991 10.1038/sj.onc.1206792

[mol212422-bib-0029] Mason KD , Carpinelli MR , Fletcher JI , Collinge JE , Hilton AA , Ellis S , Kelly PN , Ekert PG , Metcalf D , Roberts AW *et al* (2007) Programmed anuclear cell death delimits platelet life span. Cell 128, 1173–1186. 17382885 10.1016/j.cell.2007.01.037

[mol212422-bib-0030] Packer LM , Geng X , Bonazzi VF , Ju RJ , Mahon CE , Cummings MC , Stephenson SA , Pollock PM (2017) PI3K inhibitors synergize with FGFR inhibitors to enhance antitumor responses in FGFR2(mutant) endometrial cancers. Mol Cancer Ther 16, 637–648. 28119489 10.1158/1535-7163.MCT-16-0415

[mol212422-bib-0031] Pardo OE , Latigo J , Jeffery RE , Nye E , Poulsom R , Spencer‐Dene B , Lemoine NR , Stamp GW , Aboagye EO , Seckl MJ (2009) The fibroblast growth factor receptor inhibitor PD173074 blocks small cell lung cancer growth in vitro and in vivo. Can Res 69, 8645–8651. 10.1158/0008-5472.CAN-09-1576 19903855

[mol212422-bib-0032] Pearson A , Smyth E , Babina IS , Herrera‐Abreu MT , Tarazona N , Peckitt C , Kilgour E , Smith NR , Geh C , Rooney C *et al* (2016) High‐level clonal FGFR amplification and response to FGFR inhibition in a translational clinical trial. Cancer Discov 6, 838–851. 27179038 10.1158/2159-8290.CD-15-1246 PMC5338732

[mol212422-bib-0033] Pollock PM , Gartside MG , Dejeza LC , Powell MA , Mallon MA , Davies H , Mohammadi M , Futreal PA , Stratton MR , Trent JM *et al* (2007) Frequent activating FGFR2 mutations in endometrial carcinomas parallel germline mutations associated with craniosynostosis and skeletal dysplasia syndromes. Oncogene 26, 7158–7162. 17525745 10.1038/sj.onc.1210529 PMC2871595

[mol212422-bib-0034] Puthalakath H , Huang DC , O'Reilly LA , King SM and Strasser A (1999) The proapoptotic activity of the Bcl‐2 family member Bim is regulated by interaction with the dynein motor complex. Mol Cell 3, 287–296. 10198631 10.1016/s1097-2765(00)80456-6

[mol212422-bib-0035] Quispel‐Janssen JM , Badhai J , Schunselaar L , Price S , Brammeld J , Iorio F , Kolluri K , Garnett M , Berns A , Baas P *et al* (2018) Comprehensive pharmacogenomic profiling of malignant pleural mesothelioma identifies a subgroup sensitive to FGFR inhibition. Clin Cancer Res 24, 84–94. 29061644 10.1158/1078-0432.CCR-17-1172

[mol212422-bib-0036] Roberts AW , Advani RH , Kahl BS , Persky D , Sweetenham JW , Carney DA , Yang J , Busman TB , Enschede SH , Humerickhouse RA *et al* (2015) Phase 1 study of the safety, pharmacokinetics, and antitumour activity of the BCL2 inhibitor navitoclax in combination with rituximab in patients with relapsed or refractory CD20+ lymphoid malignancies. Br J Haematol 170, 669–678. 25942994 10.1111/bjh.13487 PMC4534314

[mol212422-bib-0037] Sakuragi N , A‐e Salah‐eldin , Watari H , Itoh T , Inoue S , Moriuchi T and Fujimoto S (2002) Bax, Bcl‐2, and p53 expression in endometrial cancer. Gynecol Oncol 86, 288–296. 12217750 10.1006/gyno.2002.6742

[mol212422-bib-0038] Sequist L , Cassier P , Varga A , Tabernero J , Schellens J , Delord J‐P (2014) Phase I study of BGJ398, a selective pan‐FGFR inhibitor in genetically preselected advanced solid tumors. Cancer Res 74, CT326‐326.

[mol212422-bib-0039] Sharpe R , Pearson A , Herrera‐Abreu M , Johnson D , Mackay A , Welti J , Natrajan R , Reynolds AR , Reis‐Filho JS , Ashworth A *et al* (2011) FGFR signaling promotes the growth of triple‐negative and basal‐like breast cancer cell lines both in vitro and in vivo. Clin Cancer Res 17, 5275–5286. 21712446 10.1158/1078-0432.CCR-10-2727 PMC3432447

[mol212422-bib-0040] Soria JC , DeBraud F , Bahleda R , Adamo B , Andre F , Dienstmann R , Delmonte A , Cereda R , Isaacson J , Litten J *et al* (2014) Phase I/IIa study evaluating the safety, efficacy, pharmacokinetics, and pharmacodynamics of lucitanib in advanced solid tumors. Annals Oncol 25, 2244–2251. 10.1093/annonc/mdu390 25193991

[mol212422-bib-0041] Stehbens S , Pemble H , Murrow L and Wittmann T (2012) Imaging intracellular protein dynamics by spinning disk confocal microscopy. Methods Enzymol 504, 293–313. 22264541 10.1016/B978-0-12-391857-4.00015-X PMC3495241

[mol212422-bib-0042] Stelloo E , Nout RA , Osse EM , Jurgenliemk‐Schulz IJ , Jobsen JJ , Lutgens LC , van der Steen‐Banasik EM , Nijman HW , Putter H , Bosse T *et al* (2016) Improved risk assessment by integrating molecular and clinicopathological factors in early‐stage endometrial cancer‐combined analysis of the PORTEC cohorts. Clin Cancer Res 22, 4215–4224. 27006490 10.1158/1078-0432.CCR-15-2878

[mol212422-bib-0043] Tse C , Shoemaker AR , Adickes J , Anderson MG , Chen J , Jin S , Johnson EF , Marsh KC , Mitten MJ , Nimmer P *et al* (2008) ABT‐263: a potent and orally bioavailable Bcl‐2 family inhibitor. Can Res 68, 3421–3428. 10.1158/0008-5472.CAN-07-5836 18451170

[mol212422-bib-0044] Turner N and Grose R (2010a) Fibroblast growth factor signalling: from development to cancer. Nat Rev Cancer 10, 116–129. 20094046 10.1038/nrc2780

[mol212422-bib-0045] Turner N , Lambros MB , Horlings HM , Pearson A , Sharpe R , Natrajan R , Geyer FC , van Kouwenhove M , Kreike B , Mackay A *et al* (2010b) Integrative molecular profiling of triple negative breast cancers identifies amplicon drivers and potential therapeutic targets. Oncogene 29, 2013–2023. 20101236 10.1038/onc.2009.489 PMC2852518

[mol212422-bib-0046] Turner N , Pearson A , Sharpe R , Lambros M , Geyer F , Lopez‐Garcia MA , Natrajan R , Marchio C , Iorns E , Mackay A *et al* (2010c) FGFR1 amplification drives endocrine therapy resistance and is a therapeutic target in breast cancer. Can Res 70, 2085–2094. 10.1158/0008-5472.CAN-09-3746 PMC2832818 20179196

[mol212422-bib-0047] Weeden CE , Ah‐Cann C , Holik AZ , Pasquet J , Garnier JM , Merino D , Lessene G , Asselin‐Labat ML (2018) Dual inhibition of BCL‐XL and MCL‐1 is required to induce tumour regression in lung squamous cell carcinomas sensitive to FGFR inhibition. Oncogene 37, 4475–4488. 29743589 10.1038/s41388-018-0268-2

[mol212422-bib-0048] Weiss J , Sos ML , Seidel D , Peifer M , Zander T , Heuckmann JM , Ullrich RT , Menon R , Maier S , Soltermann A *et al* (2010) Frequent and focal FGFR1 amplification associates with therapeutically tractable FGFR1 dependency in squamous cell lung cancer. Sci Translat Med 2, 62ra93. 10.1126/scitranslmed.3001451 PMC3990281 21160078

[mol212422-bib-0049] Xie L , Su X , Zhang L , Yin X , Tang L , Zhang X , Xu Y , Gao Z , Liu K , Zhou M *et al* (2013) FGFR2 gene amplification in gastric cancer predicts sensitivity to the selective FGFR inhibitor AZD4547. Clin Cancer Res 19, 2572–2583. 23493349 10.1158/1078-0432.CCR-12-3898

[mol212422-bib-0050] Yu Y , Hall T , Eathiraj S , Wick MJ , Schwartz B and Abbadessa G (2017) In‐vitro and in‐vivo combined effect of ARQ 092, an AKT inhibitor, with ARQ 087, a FGFR inhibitor. Anticancer Drugs 28, 503–513. 28240679 10.1097/CAD.0000000000000486 PMC5404396

[mol212422-bib-0051] Yuan H , Li Z‐M , Shao J , Ji W‐X , Xia W and Lu S (2017) FGF2/FGFR1 regulates autophagy in FGFR1‐amplified non‐small cell lung cancer cells. J Exp Clin Cancer Res 36, 72. 28558758 10.1186/s13046-017-0534-0 PMC5450166

