# Supplementary figures and images for: Bcl‐2 inhibitors enhance FGFR inhibitor‐induced mitochondrial‐dependent cell death in FGFR2‐mutant endometrial cancer
Source: Mol Oncol. 2019 Jan 18;13(4):738–56. doi: 10.1002/1878-0261.12422 (PMC6441928; doi:10.1002/1878-0261.12422)

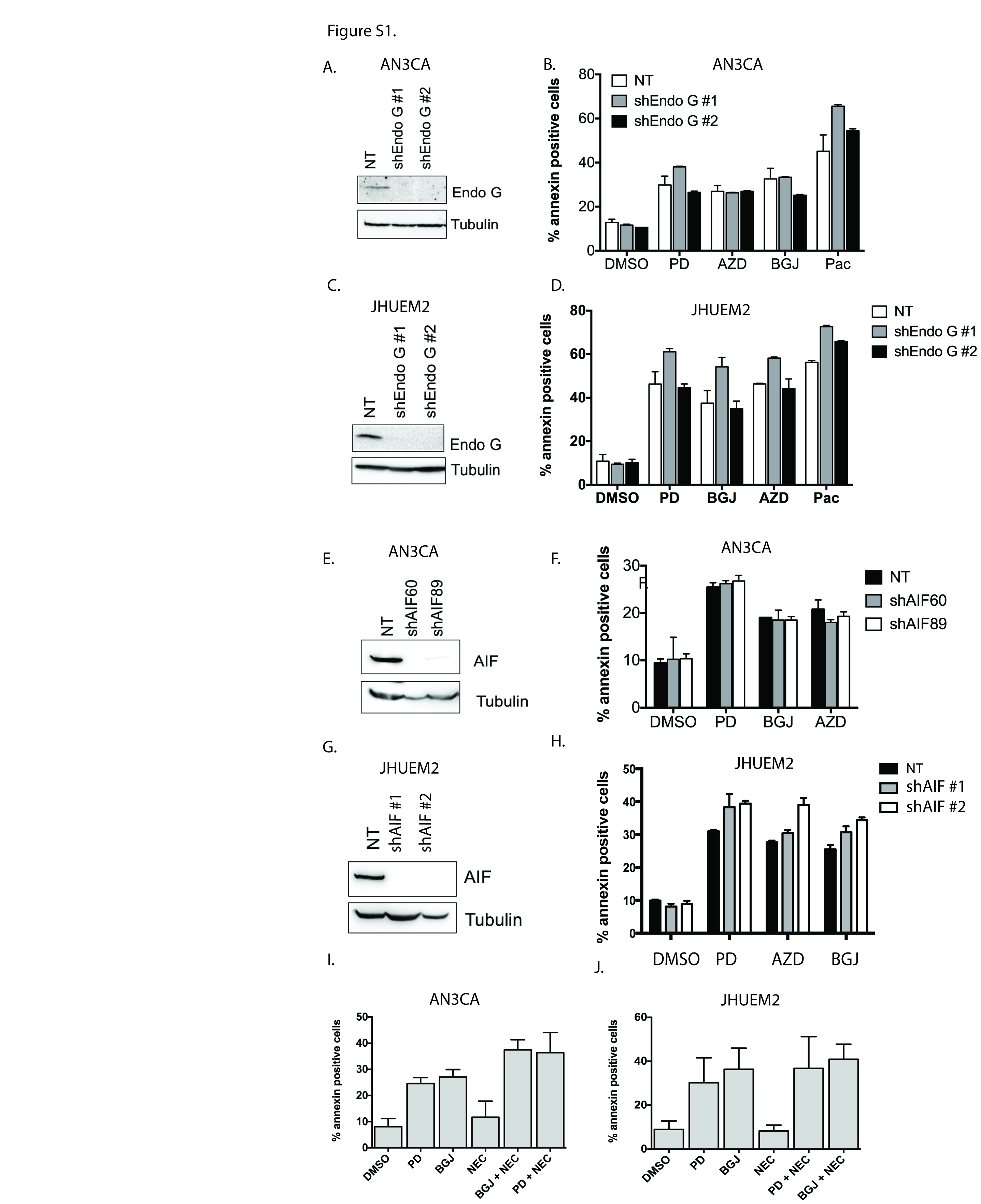

Supplement: Supplementary file 1 — Fig. S1. Western blots showing EndoG in AN3CA (A) and JHUEM2 (C) cells stably expressing nontargeting shRNA (NT), shEndoG 1 or shEndoG 2. AN3CA (B) and JHUEM2 (D) cells stably expressing NT shRNA, shEndoG 1 or shEndoG 2 were treated with 1 μm PD173074 (PD), 300 nm BGJ398 (BGJ), 300 nm AZD4547 (AZD) or 10 nm paclitaxel (Pac) for 72 h. Cell death was detected by staining cells with Annexin V. The mean percentage of Annexin V‐positive cells from three independent experiments (each performed in triplicate) is shown along with SD. Western blot showing levels of AIF in AN3CA (E) and JHUEM2 (G) cells stably expressing NT shRNA, shAIF #1 and shAIF #2. AN3CA (F) and JHUEM2 (H) cells stably expressing NT shRNA, shAIF #1 and shAIF #2 were treated with the above concentrations of PD, BGJ and AZD for 72 h. Cell death was detected by staining cells with Annexin V. The mean percentage of Annexin V‐positive cells from three independent experiments (each performed in triplicate) is shown along with SD. AN3CA (I) and JHUEM2 (J) cells were treated with 1 μm PD, 300 nm BGJ +/− 100 μm necrostatin for 72 h. Cell death was detected by staining cells with Annexin V. The mean percentage of Annexin V‐positive cells from three independent experiments (each performed in triplicate) is shown along with SD. P < 0.05 (*), <0.01 (**), <0.001 (***), <0.0001 (****). [file MOL2-13-738-s001.tif]

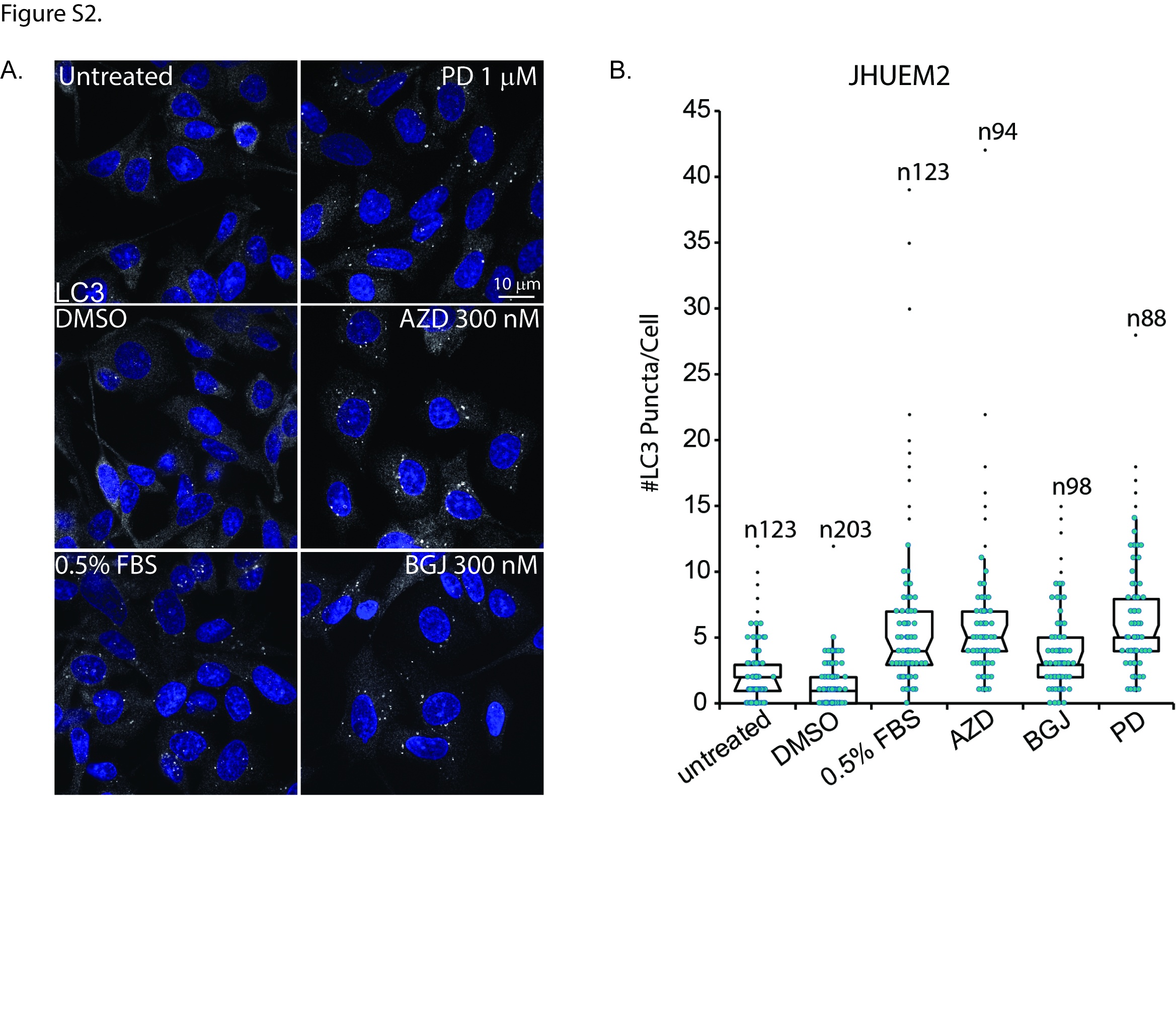

Supplement: Supplementary file 2 — Fig. S2. (A) JHUEM2 cells untreated, serum‐starved in 0.5% FBS overnight or treated with the indicated concentrations of PD173074 (PD), BGJ398 (BGJ) and AZD4547 (AZD) for 48 h prior to staining for LC3. (B) Quantitation of LC3 puncta per cell. 0.5% FBS was included as a positive control for autophagy induction. 10 fields were imaged for each condition using a 60x objective. n = per cell. Error bars show SD. Kruskal–Wallis one‐way ANOVA with a Dunn's multiple comparison test. P < 0.05 (*), <0.01 (**), <0.001 (***), <0.0001 (****). [file MOL2-13-738-s002.tif]

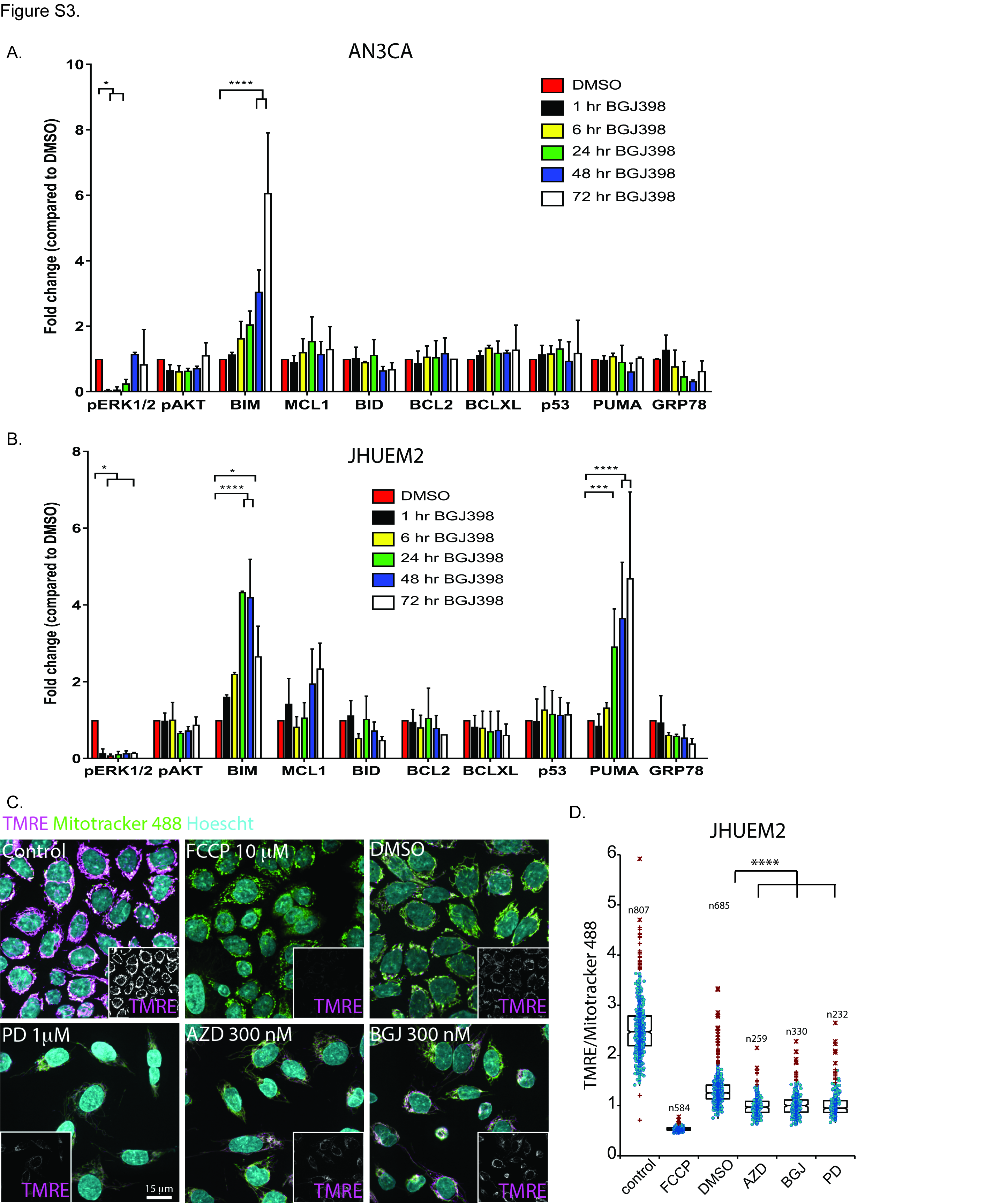

Supplement: Supplementary file 3 — Fig. S3. Densitometric analysis of AN3CA (A) and JHUEM2 (B) western blots shown in Figure 4A performed in biological triplicate. Proteins were normalized to tubulin and then expressed as a fold change of the DMSO control, with the exception of phospho‐ERK1/2 and phospho‐AKT, which were normalized to total ERK1/2 and total AKT, respectively. Error bars indicate SD. (C) JHUEM2 cells untreated (control) or treated with DMSO or the indicated concentrations of PD173074 (PD), BGJ398 (BGJ) or AZD4547 (AZD) for 48 h prior to being stained with MitoTracker Green FM and TMRE and imaged on a spinning disc microscope. Treatment of cells with 10 μm FCCP for 10 min served as a control of mitochondrial membrane depolarization. (D) Quantification of TMRE staining in JHUEM2 cells shown in (C) using a Kruskal–Wallis one‐way ANOVA with a Dunn's multiple comparison test. TMRE staining is normalized to MitoTracker staining (to indicate total mitochondrial mass) per cell. N = number of cells counted. P < 0.05 (*), <0.01 (**), <0.001 (***), <0.0001 (****). [file MOL2-13-738-s003.tif]

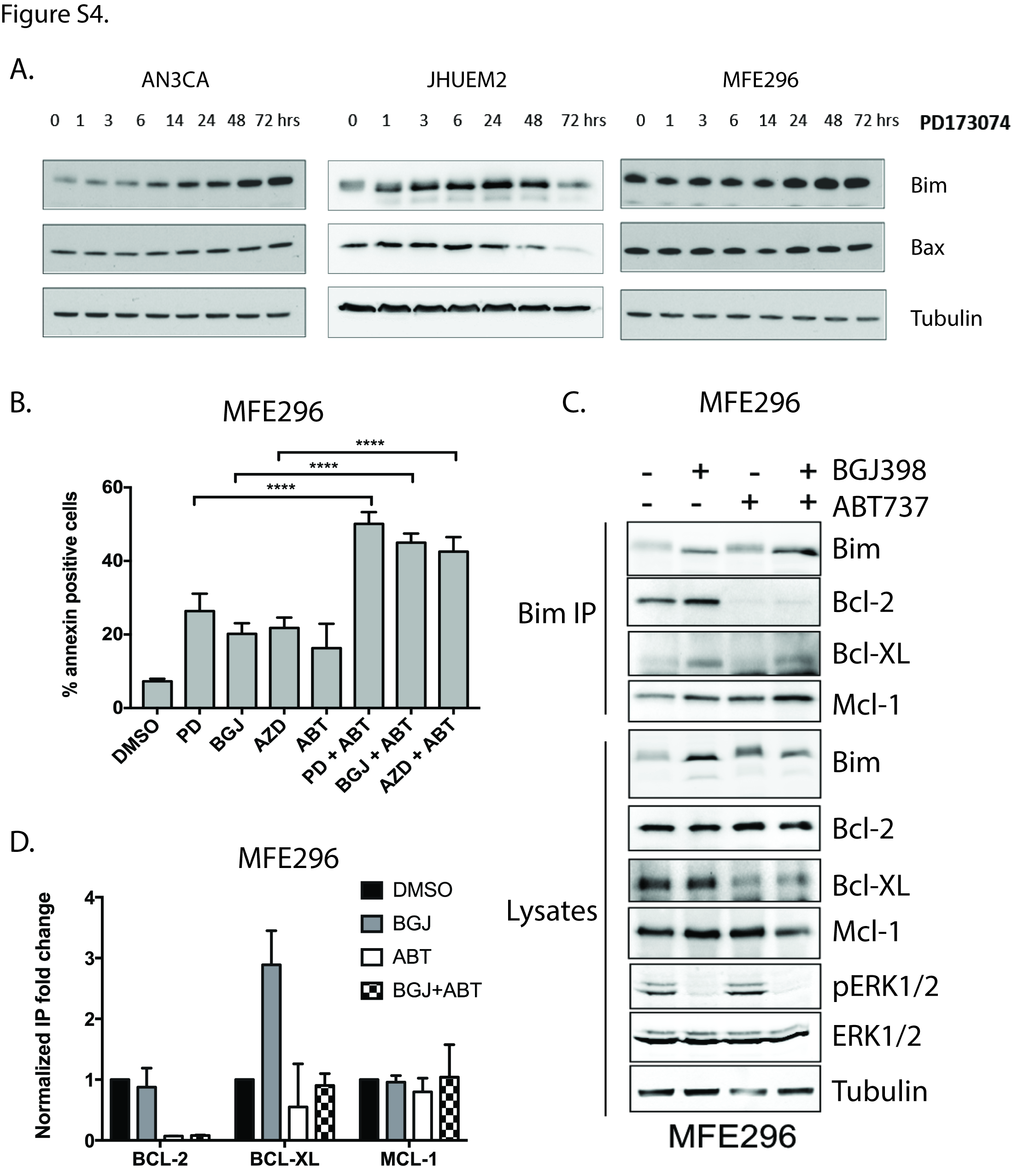

Supplement: Supplementary file 4 — Fig. S4. (A) Western blot showing AN3CA, JHUEM2 and MFE296 cells treated with 1 μm PD173074 for up to 72 h. Bim and Bax levels are shown, with tubulin as the loading control. (B) Percentage of MFE296 cells positive for Annexin V following 72‐h treatment with DMSO, 1 μm PD173074 (PD), 300 nm BGJ398 (BGJ), 300 nm AZD4547 (AZD) and 1 μm ABT737 alone or in combination. One‐way ANOVA with Dunnett's multiple comparison to compare treatments to control. Error bars show SD. P < 0.05 (*), <0.01 (**), <0.001 (***), <0.0001 (****). (C) Western blotting showing Bim, Bcl‐2, Bcl‐XL, Mcl‐1 and tubulin (loading control) in AN3CA and JHUEM2 cell lysates or Bim co‐IPs 24 h after treatment with BGJ and/or ABT737. (D) Graph showing normalized quantification of Bim co‐IPs from triplicate experiments for samples shown in (B). Error bars show SD. [file MOL2-13-738-s004.tif]
